# Natural language processing of multi-hospital electronic health records for public health surveillance of suicidality

**DOI:** 10.1038/s44184-023-00046-7

**Published:** 2024-02-14

**Authors:** Romain Bey, Ariel Cohen, Vincent Trebossen, Basile Dura, Pierre-Alexis Geoffroy, Charline Jean, Benjamin Landman, Thomas Petit-Jean, Gilles Chatellier, Kankoe Sallah, Xavier Tannier, Aurelie Bourmaud, Richard Delorme

**Affiliations:** 1 Innovation and Data unit, IT Department, Assistance Publique-Hôpitaux de Paris, Paris, France; 2 Child and Adolescent Psychiatry Department, Robert Debré University Hospital, Assistance Publique-Hôpitaux de Paris, Paris, France; 3 Département de psychiatrie et d’addictologie, GHU Paris Nord, DMU neurosciences, Bichat - Claude Bernard Hospital, Assistance Publique-Hôpitaux de Paris, 75018 Paris, France; 4 GHU Paris – psychiatry & neurosciences, 1, rue Cabanis, 75014 Paris, France; 5 NeuroDiderot, Inserm, FHU I2-D2, université Paris Cité, 75019 Paris, France; 6 CNRS UPR 3212, Institute for cellular and integrative neurosciences, 67000 Strasbourg, France; 7 Université Paris-Est Créteil, INSERM, IMRB U955 Créteil, France; 8 Service Santé Publique & URC, Hôpital Henri Mondor, Assistance Publique-Hôpitaux de Paris, Créteil, France; 9 Université Paris Cité, Paris, France; 10 URC PNVS, CIC-EC 1425, INSERM, Bichat - Claude Bernard Hospital, Assistance Publique-Hôpitaux de Paris, Paris, France; 11 Sorbonne Université, Inserm, Université Sorbonne Paris Nord, Laboratoire d’Informatique Médicale et d’Ingénierie des Connaissances pour la e-Santé (LIMICS), Paris, France; 12 Clinical Epidemiology Unit, Robert Debré University Hospital, Assistance Publique-Hôpitaux de Paris, Paris, France; 13 CIC 1426, Inserm, Paris, France; 14 Human Genetics and Cognitive Functions, Institut Pasteur, Paris, France

**Keywords:** Epidemiology, Psychiatric disorders

## Abstract

There is an urgent need to monitor the mental health of large populations, especially during crises such as the COVID-19 pandemic, to timely identify the most at-risk subgroups and to design targeted prevention campaigns. We therefore developed and validated surveillance indicators related to suicidality: the monthly number of hospitalisations caused by suicide attempts and the prevalence among them of five known risks factors. They were automatically computed analysing the electronic health records of fifteen university hospitals of the Paris area, France, using natural language processing algorithms based on artificial intelligence. We evaluated the relevance of these indicators conducting a retrospective cohort study. Considering 2,911,920 records contained in a common data warehouse, we tested for changes after the pandemic outbreak in the slope of the monthly number of suicide attempts by conducting an interrupted time-series analysis. We segmented the assessment time in two sub-periods: before (August 1, 2017, to February 29, 2020) and during (March 1, 2020, to June 31, 2022) the COVID-19 pandemic. We detected 14,023 hospitalisations caused by suicide attempts. Their monthly number accelerated after the COVID-19 outbreak with an estimated trend variation reaching 3.7 (95%CI 2.1–5.3), mainly driven by an increase among girls aged 8–17 (trend variation 1.8, 95%CI 1.2–2.5). After the pandemic outbreak, acts of domestic, physical and sexual violence were more often reported (prevalence ratios: 1.3, 95%CI 1.16–1.48; 1.3, 95%CI 1.10–1.64 and 1.7, 95%CI 1.48–1.98), fewer patients died (*p* = 0.007) and stays were shorter (*p* < 0.001). Our study demonstrates that textual clinical data collected in multiple hospitals can be jointly analysed to compute timely indicators describing mental health conditions of populations. Our findings also highlight the need to better take into account the violence imposed on women, especially at early ages and in the aftermath of the COVID-19 pandemic.

## Introduction

Since its outbreak, the COVID-19 pandemic raised concerns about the heavy toll it would take on mental health. Clinicians and policymakers strived to rapidly identify populations that were the most mentally at-risk in order to judiciously design prevention campaigns and optimally allocate scarce resources. In particular, they feared a raise of the incidence of suicide attempts (SA)^1^. More than 3 years after the pandemic outbreak, strong evidence has been collected that confirmed the well-foundedness of these concerns^2^, but the first studies were either inconclusive or contradictory^3–5^. Moreover, only recently were youths, especially girls, unambiguously identified as being the most affected group^6–10^. The underlying vulnerability mechanism inducing this sex- and age-dependent dynamics are still poorly understood although a possible impact of the pandemic context on some risk factors such as child abuse has been suggested^11^. The time required for evidence collection impeded the adoption of timely and targeted measures to mitigate the impact of the pandemic on these populations. New tools are consequently required to better monitor the mental health of populations and facilitate the management of upcoming crises^12,13^.

Prospects in that direction are opened by the collection in various databases of ever more naturalistic data that reflect the mental health conditions of populations, in particular textual data, and by the advent of natural language processing (NLP) algorithms to analyse them automatically and timely. Indeed, new NLP technologies increasingly enable the computation of structured variables using large text corpora that were primarily generated for another objective (e.g., for care, by users of social media, etc.). Algorithms based on artificial intelligence such as neural networks have achieved impressive performances on various tasks related to the analysis of human language, including in clinical applications^14,15^.

Until now, most studies that pursued this line of enquiry have focused on social media data^16–19^. Although they detected impacts of the COVID-19 outbreak on contents posted on these media, it remained unclear whether concrete guidelines for clinicians and policymakers could be deduced from these observations. Indeed, information collected using this media concerned only a few outcomes of interest whose reporting moreover depends on its perceived importance by the population. Moreover, contents posted on social media neither covered evenly the population of interest, over- or under-representing subgroups of different demographic and social conditions, nor allowed proper stratifications to identify populations at-risk. Applying NLP algorithms on data collected in hospitals’ electronic health records (EHR) instead of social media appears as a promising field of research to circumvent these limitations. Specific technical and methodological difficulties, however, have to be overcome^20–22^.

First, although some projects have already demonstrated that NLP algorithms applied on clinical reports could extract variables of interest regarding mental health^23–27^, many of these algorithms were developed using disease-, age- or hospital-specific cohorts and concerns have been raised regarding their generalisability^28^. It is therefore necessary to train and validate NLP algorithms on datasets that are representative of various contexts of care to detect indications on mental health that are often scattered among millions of reports edited throughout care organisations. Second, platforms allowing for the analysis of EHR often encompass data collected in a single hospital that may not be sufficient to monitor mental health of populations at a regional or national level. Privacy and technical issues limit the sharing of data or algorithms among platforms, and developing any multi-hospital indicator may consequently require a costly and complex replication of developments. Even when data from multiple hospitals are available on a single platform, their aggregation in a joint analysis is rarely straightforward as local specificities shall still be accounted for to avoid biases.

We aim at demonstrating that mental health indicators can be computed timely at a population level analysing jointly millions of clinical reports collected in multiple hospitals. We therefore focused on a specific use case, the monitoring of suicidality during the COVID-19 crisis. First, we developed and validated new NLP algorithms that measured our main indicator, i.e., the monthly number of hospitalised SA in fifteen hospitals of the Paris area. Second, we conducted a retrospective study considering this indicator both before and after the COVID-19 outbreak and assessed whether we detected the now-established surge of SA among youths and especially girls. Third, we conducted an exploratory analysis to estimate whether further information on the underlying mechanisms inducing this surge could be obtained by detecting risk factors mentioned in clinical reports.

## Results

### Cohort of hospitalisations caused by SA

From August 1st, 2017 until June 31st, 2022, we included in our analysis 2,911,920 hospitalisations, gathering 14,023 hospitalisations linked to SA as classified by our main, hybrid NLP algorithm (0.5% of considered hospitalisations, related to 11,786 independent individuals). We observed less stays before than after the COVID-19 outbreak [5954 (42.5%) and 8069 (57.5%), respectively] (Table 1). The mean age at admission was 38.0 years (20.7 SD). When considering sex-ratio distribution along the observation period of our study, we observed that females counted approximately for two thirds of SA-caused stays (9015, 64.3%) and males for one third of them (5008, 35.7%).

**Table 1:** Number of hospitalisations caused by suicide attempts, seasonally-adjusted long-term means and trends, and post-pandemic trend variations.

		No. hospitalisations caused by suicide attempts (%) - pre-pandemic period	No. hospitalisations caused by suicide attempts (%) - post-pandemic period	Long term mean ${\alpha }_{0}$ (95%CI)^a^	Long term trend ${\alpha }_{1}$(95%CI)^a^	Trend variation after COVID-19 outbreak ${\alpha }_{2}$(95%CI)^a^
Male	8–17	232 (3.9%)	281 (3.5%)	6.0 (4.4–7.7)	0.1 (−0.0–0.1)	0.1 (−0.1–0.2)
	18–25	271 (4.6%)	405 (5.0%)	6.5 (3.9–9.0)	0.1 (−0.0–0.3)	0.1 (−0.1–0.4)
	26–65	1451 (24.4%)	1755 (21.7%)	39.7 (34.4–45.0)	0.3 (0.0–0.6)	0.3 (−0.2–0.9)
	66–	300 (5.0%)	313 (3.9%)	10.2 (8.0–12.4)	−0.1 (-0.2–0.0)	0.3 (0.1–0.6)
	All Ages	2254 (37.9%)	2754 (34.1%)	62.4 (55.6–69.2)	0.4 (0.0–0.8)	0.9 (0.2–1.6)
Female	8–17	865 (14.5%)	1535 (19.0%)	23.2 (16.8–29.5)	0.1 (−0.2–0.5)	1.8 (1.2–2.5)
	18–25	549 (9.2%)	1045 (13.0%)	13.5 (9.2–17.8)	0.2 (−0.0–0.4)	1.1 (0.7–1.5)
	26–65	1810 (30.4%)	2197 (27.2%)	44.8 (38.4–51.3)	0.7 (0.4–1.0)	−0.2 (−0.8–0.5)
	66–	476 (8.0%)	538 (6.7%)	14.0 (10.6–17.3)	0.1 (−0.1–0.2)	0.1 (−0.3–0.4)
	All Ages	3700 (62.1%)	5315 (65.9%)	95.5 (83.2–107.7)	1.1 (0.4–1.7)	2.8 (1.6–4.1)
Overall		5954 (100.0%)	8069 (100.0%)	157.9 (142.0–173.7)	1.5 (0.6–2.3)	3.7 (2.1–5.3)

^a^Parameters were computed by ordinary least squares regressions following Eq. (1). All models were controlled for seasonal effects. 95%CIs were based on standard errors. Number of hospitalisation stays caused by suicide attempts were used as dependent variables.

### NLP algorithms

As shown in Table 2, the positive predictive value (PPV) of the main hybrid algorithm for SA detection (0.85) was superior to the PPV of an alternative rule-based algorithm (0.51). Interestingly, NLP algorithms featured constant PPV before and after the COVID-19 outbreak for the detection of both SA-caused stays and risk factors. The inter-annotator positive and negative agreements were [0.92;0.5] for SA detection, [1.0;1.0] for four of the algorithms detecting risk factors (i.e., history of SA, physical, sexual, and domestic violence) and [1.0;-] for the algorithm detecting social isolation (i.e., no false positive detection was observed).

**Table 2:** Positive predictive values of the natural language processing algorithms.

	Pre-pandemic positive predictive value (95%CI, No. annotated records)^a^	Post-pandemic positive predictive value (95%CI, No. annotated records)^a^
Main algorithm used to detect hospitalisations caused by suicide attempts (hybrid machine learning and rule-based approach)	0.85 (0.76–0.91, 85)	0.86 (0.76–0.92, 77)
Alternative algorithm used to detect hospitalisations caused by suicide attempts (rule-based approach)	0.51^b^	0.52^b^
Algorithm used to detect social isolation risk factor	1.00^c^ (0.89–1.00, 30)	0.96^c^ (0.79–0.99, 23)
Algorithm used to detect domestic violence risk factor	0.96^c^ (0.79–0.99, 23)	0.96^c^ (0.80–0.99, 24)
Algorithm used to detect sexual violence risk factor	0.84^c^ (0.70–0.93, 38)	0.96^c^ (0.82–0.99, 28)
Algorithm used to detect physical violence risk factor	0.86^c^ (0.69–0.95, 29)	0.96^c^ (0.82–0.99, 28)
Algorithm used to detect suicide attempt history risk factor	0.93^c^ (0.78–0.98, 29)	0.83^c^ (0.66–0.93, 30)

^a^Wilson score intervals were used to compute the 95%CIs.

^b^For the estimation of the performances of the alternative rule-based SA-classification algorithm, additional stays were drawn randomly among the stays that were classified as SA-caused by the rule-based algorithm but not by the hybrid algorithm—see Supplement. The Wilson score intervals could not be computed using this method.

^c^For the estimation of the performances of the risk factors algorithms, stays were drawn randomly among those that were labelled positive both by the main SA-classification algorithm and by the risk factors-classification algorithms.

### Mental health indicators

**Fig. 1:**
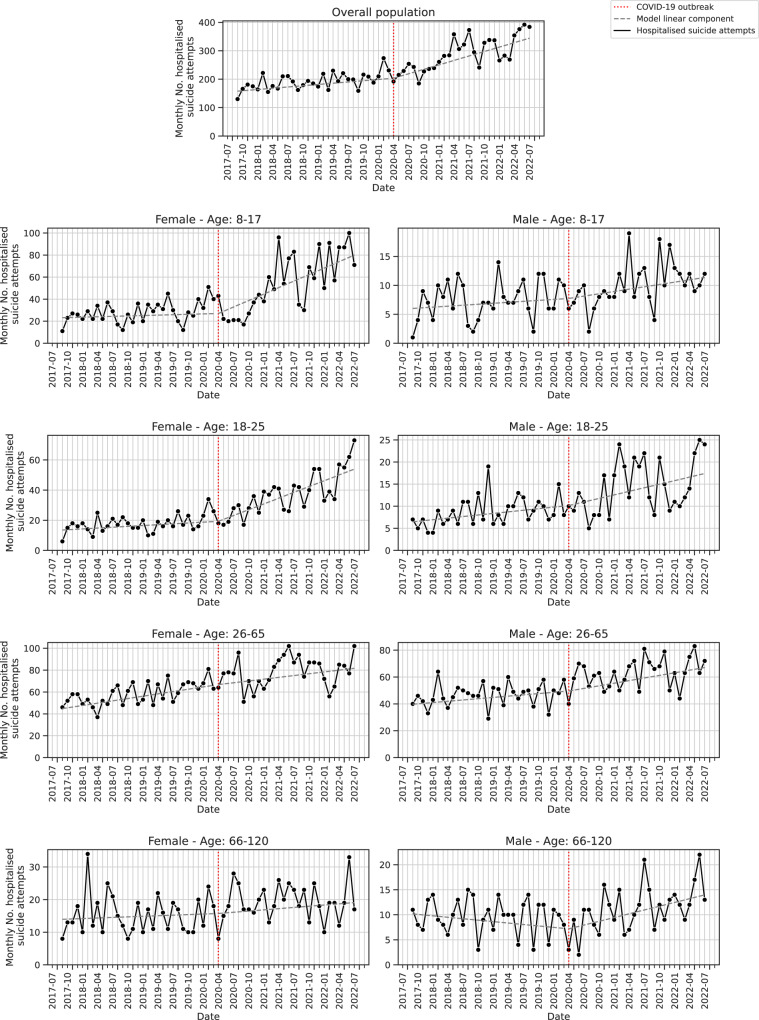
Monthly number of hospitalised suicide attempts.

Figure 1 shows the sex- and age-stratified time-series of the monthly number of hospitalised SA. We noticed a modification of SA dynamics after the COVID-19 outbreak with the slope variation indicating a statistically significant increase of SA affected the overall population (3.7, 95%CI 2.1–5.3). This effect was mainly driven by girls aged 8–17 (1.8, 95%CI 1.2–2.5) and young women aged 18–25 (1.1, 95%CI 0.7–1.5), and marginally by men (0.9, 95%CI 0.2–1.6). The residuals did not feature any noticeable time trend indicating that temporal variations were correctly accounted for by the linear, seasonally-adjusted model (Supplementary Figs. 1, 2).

### Sensitivity analysis

As shown in Supplementary Figs. 3–13 and Supplementary Tables 1–3, four sensitivity analyses confirmed the robustness of the results we reported: they were either still present (analysis with an alternative rule-based NLP algorithm, adjusting for a potential completeness-induced bias or conducting per-hospital analysis) or not significant (analysis with an alternative claim-based algorithm). Considering single hospitals often led to results that were not statistically significant, highlighting the interest of considering jointly multiple hospitals.

### Characteristics of SA-caused hospitalisations

The variety and proportion of methods used to attempt suicide were similar across time but featured a stronger amount of intentional drug overdose among women (Supplementary Fig. 14). The time-to-exit indicated shorter stays after the COVID-19 outbreak and the survival analysis showed that stays ended less often by patient’s death ($p\le 0.001$ and $p=0.007$, respectively). Kaplan-Meier curves indicated that short stays were on average shorter after COVID-19 outbreak while the duration of longer stays remained unaffected (see Supplementary Fig. 15).

### Exploratory analysis of risk factors

**Fig. 2:**
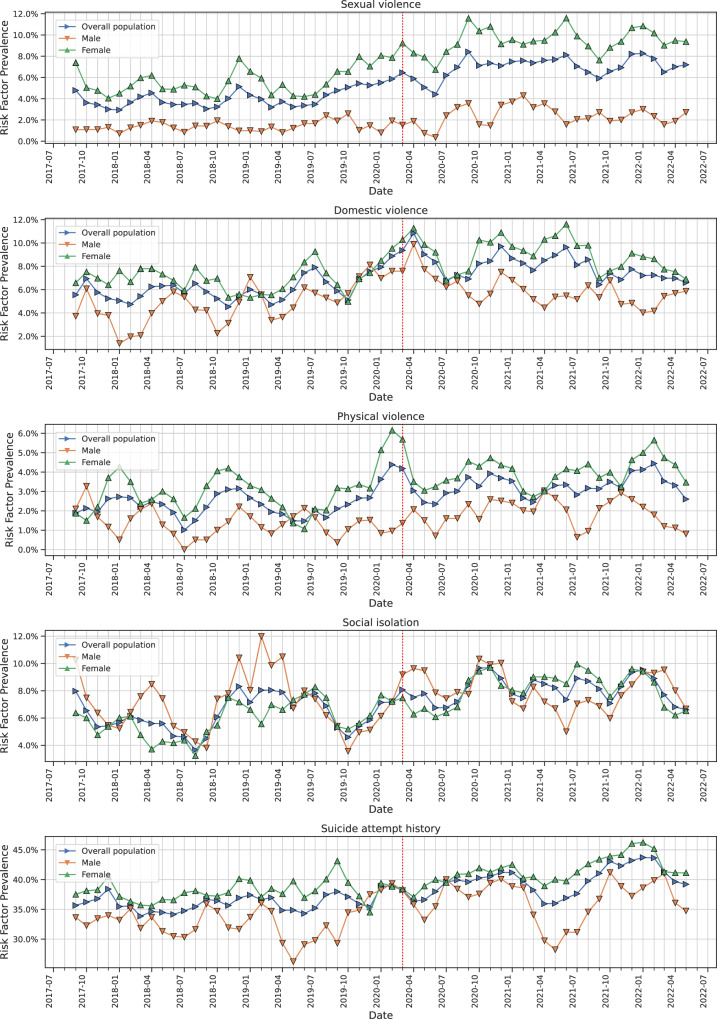
Monthly prevalence of risk factors reported in discharge summaries of hospitalised suicide attempts. *A centred 3-month averaging was applied to smooth the curves*.

Finally, we explored how known SA risk factors were reported by clinicians in discharge summaries of SA-caused stays. Whereas personal suicide attempt history and social isolation were equally mentioned for males and females, acts of domestic, physical and sexual violence were more often reported for females both before and after the outbreak. However, the prevalence of reported risk factors evolved during the study period we examined (Fig. 2). We observed a strong increase of any kind of violence after COVID-19 outbreak (prevalence ratios: 1.3, 95%CI 1.16–1.48; 1.3, 95%CI 1.10–1.64 and 1.7, 95%CI 1.48–1.98 for domestic, physical and sexual violence, respectively - see Table 3). Interestingly, we only observed a marginal effect of the COVID-19 outbreak on the prevalence of personal suicide attempt history and social isolation (prevalence ratio 1.1, 95%CI 1.05–1.14; 1.2, 95%CI 1.09–1.39, respectively).

**Table 3:** Prevalence of risk factors in pre- and post-pandemic periods.

Risk factor	Group	Pre-covid prevalence (number of stays)	Post-covid prevalence (number of stays)	Prevalence ratio (95% CI)^a^	*p*-value^a^
Domestic violence	Overall	0.06 (360)	0.08 (639)	1.3 (1.16–1.48)	**<0.0001**
	Male	0.05 (106)	0.06 (163)	1.3 (0.99–1.60)	0.059
	Female	0.07 (254)	0.09 (476)	1.3 (1.13–1.51)	**0.00035**
Physical violence	Overall	0.02 (142)	0.03 (258)	1.3 (1.10–1.64)	**0.0047**
	Male	0.01 (29)	0.02 (50)	1.4 (0.90–2.22)	0.14
	Female	0.03 (113)	0.04 (208)	1.3 (1.02–1.60)	**0.032**
Sexual violence	Overall	0.04 (244)	0.07 (567)	1.7 (1.48–1.98)	**<0.0001**
	Male	0.01 (31)	0.02 (66)	1.7 (1.14–2.66)	**0.0098**
	Female	0.06 (213)	0.09 (501)	1.6 (1.40–1.91)	**<0.0001**
Social isolation	Overall	0.06 (383)	0.08 (640)	1.2 (1.09–1.39)	**0.00071**
	Male	0.07 (159)	0.08 (216)	1.1 (0.91–1.35)	0.31
	Female	0.06 (224)	0.08 (424)	1.3 (1.13–1.54)	**0.00049**
Suicide attempt history	Overall	0.36 (2160)	0.40 (3199)	1.1 (1.05–1.14)	**<0.0001**
	Male	0.33 (749)	0.36 (993)	1.1 (1.00–1.17)	**0.037**
	Female	0.38 (1411)	0.42 (2206)	1.1 (1.03–1.15)	**0.0013**

^a^*p* values were computed using the Fisher exact test. The normal approximation method was used to obtain the 95%CIs.

Bold values identify statistical significance (*p* ≤ 0.05).

## Discussion

### Retrospective validation of indicators related to suicidality

The aim of our study was to demonstrate that surveillance indicators describing mental health of populations could be computed analysing jointly clinical reports edited in multiple hospitals. We therefore developed and applied NLP algorithms on data collected retrospectively during an observation period of 5 years which encompassed approximately three million hospital stays. We assessed whether this methodology could detect known variations in the dynamics of SA in children and adults after the COVID-19 outbreak and provide information on the mechanisms underlying this phenomenon. We identified a large-scale sample of 14,023 SA-caused hospitalisations during the whole period covered by the analysis. We observed an increase in the monthly number of hospitalised SA after the COVID-19 outbreak, especially among females aged 8–25 years old in accordance with previous findings^2,6,7,9^. Interestingly, we detected that violence -which was known as a risk factor of SA that strongly affects girls and young women-^29,30^ was more frequently reported in the discharge summaries of SA-caused stays after the pandemic outbreak, stressing its role in the atypical dynamic of SA in this population.

### Impact of the COVID-19 context and violence on mental health

The detected associations highlight retrospectively the major impact of the COVID-19 pandemic on women’s mental health. Although a direct link between lockdown and violence imposed on females was not established in our study, our findings emphasised that this period was critical for women’s safety and resulted in an exacerbation of SA in the immediate aftermath of the pandemic^31–33^. According to the World Health Organization (WHO), violence affects one in three women worldwide, who are domestically, physically or sexually intimidated^34^. The impact of violence is major on mental health of girls and women, but more generally alters their health by increasing their risk at long-term of diabetes, chronic pain, and cardiovascular disease among others^35,36^. Actions to prevent SA among girls and young women must include measures to protect them from violence: challenging discriminatory gender norms and attitudes that condone violence against women, reforming discriminatory family laws, promoting women’s access to gainful employment and secondary education, reducing exposure to violence during childhood, and addressing substance abuse^37,38^. If our methodology were applied during the COVID-19 crisis, the computed indicators would have raised an early alert helping thus clinicians and policymakers to mitigate the impact of the pandemic on the mental health of girls.

### NLP to study mental health of populations

Our study validated retrospectively the feasibility of analysing automatically EHR contained in multi-hospital clinical data warehouses to provide epidemiological insights on mental health of populations and early identify at-risk groups during crises. Using NLP algorithms allowed us to summarise data collected in millions of reports without introducing additional constraints in clinical practices. We confirmed that NLP algorithms could efficiently detect SA and some of its known risk factors^24–26^, showing that we could both detect rare events among hospitals’ EHRs and circumvent the limited completeness of claim data^39^. Even better, the adoption of a hybrid architecture that contained artificial intelligence components improved the algorithms’ performances compared to purely rule-based approaches^25^. The statistical analysis relying on data provided by NLP algorithms was robust to technical and methodological choices, indicating in particular that concerns about the reliability of indicators computed using multi-hospital EHR could be addressed efficiently. In the future, applying prospectively these algorithms could allow us to compute real-time mental health indicators that would complement already available surveillance epidemiological tools. The methodology of this study could moreover be extended to further process clinical notes using NLP algorithms, for instance to extract information related to the consumption of care (medications, previous visits, etc.) or to socioeconomic determinants (unemployment, dwelling type, etc.). NLP algorithms could moreover be implemented in a clinical setting to better target the prevention of SA^40^.

### Limitations

This study has several limitations. First, it was an observational study that was not designed to test causality. Second, we conducted a retrospective study that should be extended by a prospective study to fully demonstrate the usefulness of the developed mental health indicators to address upcoming crises. Third, we detected SA and risk factors analysing their reporting by clinicians, but this reporting may depend on varying clinical practices, experience of physicians, ease of use of the EHR, etc. Fourth, visits to emergency departments that were not followed by hospitalisations were discarded due to the current availability of emergency reports in the database, thus limiting our analysis to the most severe SA resulting in a hospitalisation stay and reducing the exploration of the whole severity spectrum of SA.

## Conclusions

In conclusion, we demonstrated that naturalistic data collected in the EHR of multiple hospitals, both structured and unstructured, could be leveraged to compute indicators describing mental health conditions of populations. To achieve this, we analysed retrospectively millions of clinical reports using NLP algorithms to identify populations whose suicidality was the most affected during a critical period, the COVID-19 pandemic. We detected some risk factors associated with the variation of the number of severe SA being hospitalised. Our results also highlighted the need to better take into account violence imposed on women, especially at early ages, to prevent the occurrence of severe SA in this at-risk group.

## Methods

This study followed the REporting of studies Conducted using Observational Routinely-collected health Data (RECORD) reporting guideline (checklist available in the Supplement)^41^. The methods were performed in accordance with relevant guidelines and regulations and approved by the institutional review board of the Greater Paris University Hospitals (IRB00011591, decision CSE 21-13). French regulation does not require the patient’s written consent for this kind of research but in accordance with the European General Data Protection Regulation the patients were informed and those who opposed the secondary use of their data for research were excluded from the study. Data was pseudonymised by replacing names and places of residence by aliases.

### Choice of primary measure

We chose as primary measure the monthly number of hospitalisations caused by SA. Since the seminal work of Durkheim^42^, suicidality is indeed considered as a compelling marker of a population’s mental health. Many studies have been dedicated to the description of its variation with time and among subgroups, in particular when mentally at-risk populations needed to be identified (e.g., in the aftermath of economic crises or infectious epidemics)^43,44^. Some of these studies focused on completed suicide attempts but this measure has limitations that makes it less suited for the monitoring of the mental health of populations as, (i) the reporting of SA on death certificates may be poorly reliable and is often delayed, (ii) and only a few events can be observed for some at-risk populations such as youths. Our choice to focus on suicide attempts increased the number of observed events and allowed us to leverage high-quality and timely data collected within EHRs.

### Study design, setting, and participants

We conducted a multicentre observational retrospective cohort study. We considered hospitalisation stays caused by SA defined as a non-fatal self-directed potentially injurious behaviour with any intent to die as a result of the behaviour^7^. This definition discarded hospitalisations that only mentioned self-harm or suicide ideation. We considered data collected in the EHR of all patients hospitalised between August 1st, 2017 and June 31st, 2022 in the AP-HP (Assistance Publique-Hôpitaux de Paris) university hospitals. We selected only adult and paediatric hospitals for which the deployment of the EHR ensured a temporal stability of data collection during the study period (*n* = 15, see Supplementary Table 4 and Supplementary Fig. 16).

We examined administrative data (age, sex, dates of stay, death during stay), diagnoses issued from claim data (coded using the International Classification of Diseases 10th revision) and clinical reports. All reports were considered at the screening stage but only the last-edited discharge summary of each stay was used by the stay-classification algorithm.

To identify which stays were relative to a SA, we first detected patients whose clinical reports contained at least one keyword relative to SA (screening stage, see Supplementary Fig. 17 and Supplementary Table 5). Second, to discard the numerous false positive detections of the first stage we applied a stay-classification algorithm based on artificial intelligence. Third, we discarded patients who were aged under eight at admission. We chose this intermediate threshold as the intentionality of a SA is difficult to establish for very young children but SA are nevertheless reported by clinicians before the age of ten. Finally, if two SA-caused hospitalisations occurred within a period of 15 days for the same patient, we considered only the first occurrence to avoid spurious multiple counting.

### Data sources

AP-HP comprises 38 hospitals spread across the Paris area (gathering more than 22,000 beds). Data collected in the EHR software and in the claim database are merged together in the AP-HP clinical data warehouse on a daily and monthly basis, respectively. The research database follows the Informatics for Integrating Biology & the Bedside standard^45^. Data was extracted on July 4th, 2022.

### Development and validation of natural language processing algorithms

At the screening stage, a dictionary of keywords relative to SA and grouped by modality was looked for in the clinical reports (e.g., “suicide attempts by jumping from height”, “intentional drug overdose” as well as orthographic variations of these terms, see Supplementary Table 5). At the stay-classification stage, keywords were extended as regular expressions (i.e., sequences of characters that define a search pattern) and looked for among the discharge summaries of the screened patients. Each detected mention was classified as valid (i.e., a stay really caused by a SA) or invalid using a NLP algorithm that followed the RoBERTa neural network architecture (see Supplementary Fig. 18)^46^. It detected false detections of SA-caused by negative mentions, mentions not relative to the patient, relative to the patient’s history, expressed as reported speech or as an hypothesis. A stay was classified as SA-caused if at least one valid mention of SA was detected in its discharge summary. The NLP algorithm was developed using *EDS-NLP* v0.6.1^47^.

The dataset was divided by hospital into training (*n* = 10) and validation (*n* = 5) sets (see Supplementary Table 4). The SA dictionary was initiated before accessing data by a group of four expert senior clinicians (V.T., P.-A.G., B.L., R.D.) and used to screen a first set of eligible stays. Then, 465 eligible stays were randomly selected among the training set and were annotated by authors. Each SA-keyword found in the documents was labelled as a true or false detection of a SA mention and each SA mention was qualified when relevant as *negation, relative to a family member, relative to patient history, reported speech, hypothesis* (1,571 mentions labelled, see the Annotation Guidelines in the Supplement). New SA-keywords detected during annotation were added to the dictionary. The annotations were then used to train the neural network that was initialised with a CamemBERT language model pre-trained on 21 million clinical reports written in French^48,49^. The PPV of the algorithm used to detect SA-caused stays was assessed by a chart review by two expert clinicians (V.T., B.L.) of a sample of 162 discharge summaries randomly drawn from the validation set and divided in pre- and post-pandemic periods. Among them, 15 stays were blindly annotated by the two clinicians to measure inter-annotator agreement (positive and negative agreements)^50^. The sensitivity of the algorithm could not be measured as SA-caused hospitalisations represented a tiny proportion of the hospitalisation stays and a massive dataset should consequently be annotated to estimate it.

### Variables

We collected the following variables for each SA-caused stay: age at admission, sex, admission date, length of stay, death during the stay, known SA risk factors reported in discharge summaries [social isolation, domestic, sexual & physical violence and personal suicide attempt history (see Supplementary Table 6). Risk factors were detected in the last-edited discharge summaries of SA-caused stays using rule-based NLP algorithms (see Supplement)], claim diagnostic codes, hospital location.

### Statistical analysis

Continuous variables were reported as means with standard deviations (SD). Qualitative variables were reported as numbers (%). To estimate the impact of the COVID-19 pandemic on the dynamics of SA-caused hospitalisations we conducted a single-group interrupted time-series analysis^51^. We considered the monthly numbers of SA-caused hospitalisations ${N}_{T}$ divided into a pre-pandemic period (after August 1st, 2017 and before February 29th, 2020) and a post-pandemic period (after March 1st, 2020 and before June 31st, 2022). We adjusted for the mean, the long-term trend and the seasonality^52^, and tested the presence of a post-pandemic variation of the trend using the following equation:

1 $${N}_{T}={\alpha }_{0}+{\alpha }_{1}T+{\alpha }_{2}(T-\tilde{T})\times 1\{T \ge \tilde{T}\}+\mathop{\sum }\limits_{m=Jan}^{Dec}\,{\beta }_{m}\times 1\{T=m\}+{e}_{T}$$

with $T$ a running counter of months since August, 2017; $\widetilde{T}$ the date of the pandemic outbreak (March 2020); ${\alpha }_{0}$ and ${\alpha }_{1}$ parameters characterising respectively the mean and the long-term trend; ${\alpha }_{2}$ the trend variation after the COVID-19 outbreak; ${\beta }_{m}$ parameters adjusting for seasonal variations with $m$ standing for months from January to December, and ${e}_{T}$ a random error. We estimated the model and confidence intervals (CI) via ordinary least-squares regression.

We conducted a subgroup analysis according to sex and age (8–17, 18–25, 26–65 and 66–). To compare the severity of SA-caused hospitalisations in the pre- and post-pandemic periods, accounting for the potential bias induced by the censoring of non-terminated stays, we considered length of stay as censored data and compared groups using log-rank tests. The same strategy was applied for the stays that ended with the patient’s death.

In an exploratory analysis we compared the pre- and post- pandemic prevalence ratios of the reported risk factors using Fisher’s exact tests. All tests were 2-sided and *p*-values were considered statistically significant when $\le 0.05$. All estimations were reported with their 95% CI. Statistical analysis was performed using the *statsmodels* v0.13.2 and *lifelines* v0.26.4^53,54^.

### Sensitivity analysis

We conducted four sensitivity analyses and assessed whether the statistically significant effects detected in the main analysis were still consistent in these sub-analyses (see Supplement for details): (i) we considered an alternative stay-classification algorithm that used only diagnosis claim codes; (ii) we evaluated an alternative stay-classification algorithm replacing the neural network by a rule-based NLP algorithm that did not rely on machine learning (we estimated the algorithm’s PPV annotating additional records); (iii) we adjusted for potential bias induced by missing data by dividing each monthly number of SA by the average completeness of discharge summaries^55^; (iv) we examined each hospital separately.

### Supplementary information

RECORD_Checklist

Supplementary Information

## Data Availability

Access to the clinical data warehouse’s de-identified raw data can be granted following the process described on its website: www.eds.aphp.fr. A prior validation of the access by the local institutional review board is required. In the case of non-AP-HP researchers, the signature of a collaboration contract is moreover mandatory. The analysis code used to conduct this analysis is made freely available with publication.

## Code availability

The underlying code for this study is available in 10.5281/zenodo.8283122 and can be accessed via this link https://github.com/aphp-datascience/study-nlp-suicidality-surveillance. The parameters of the machine learning model are not published due to privacy considerations, but they are available upon request for other projects conducted on the secure platform of the AP-HP’s clinical data warehouse on condition of a prior validation by the institutional review board.
