# Supplementary material for: Natural language processing of multi-hospital electronic health records for public health surveillance of suicidality
Source: Npj Ment Health Res. 2024 Feb 14;3:6. doi: 10.1038/s44184-023-00046-7 (PMC10955903; doi:10.1038/s44184-023-00046-7)
Supplement: Supplementary file 2 — Supplementary Information [file 44184_2023_46_MOESM2_ESM.pdf]

Online Supplement:

Natural language processing of multi-hospital electronic health records for public health surveillance of suicidality

List of contents 2
Model's residuals 3
Sensitivity analysis 5
Modalities of suicide attempt 19
Severity analysis 21
Details on cohort creation 22
Details on the algorithms 26
Annotation guidelines 36
References 39

List of contents

Supplementary Figure1. Detected and modelled monthly numbers of suicide attempts
Supplementary Figure2. Model's residuals
Supplementary Figure3. Monthly numbers of hospitalisations caused by suicide attempts - rule-based algorithm
Supplementary Table1. Numbers and modelling of hospitalisations caused by suicide attempts - rule-based algorithm
Supplementary Figure4. Monthly numbers of hospitalisations caused by suicide attempts - claim based algorithm
Supplementary Table2. Numbers and modelling of hospitalisations caused by suicide attempts - claim based algorithm
Supplementary Figure5. Monthly numbers of hospitalisations caused by suicide attempts - completeness-adjusted
Supplementary Table3. Numbers and modelling of hospitalisations caused by suicide attempts - completeness-adjusted
Supplementary Figure6. Per-hospital forest plot of trend variations - overall population
Supplementary Figure7. Per-hospital forest plot of trend variations - girls
Supplementary Figure8. Per-hospital forest plot of trend variations - overall population, rule-based algorithm
Supplementary Figure9. Per-hospital forest plot of trend variations - girls, rule-based algorithm
Supplementary Figure10. Per-hospital forest plot of trend variations - overall population, completeness-adjusted
Supplementary Figure11. Per-hospital forest plot of trend variations - girls, completeness-adjusted
Supplementary Figure12. Per-hospital forest plot of trend variations - overall population, claim-based algorithm
Supplementary Figure13. Per-hospital forest plot of trend variations - girls, claim-based algorithm
Supplementary Figure14. Monthly proportions of each modality of suicide attempt
Supplementary Figure15. Kaplan-Meier curves relative to stay duration and death during stay
Supplementary Table4. Hospitals of the Greater Paris University Hospitals considered in this study
Supplementary Figure16. Per-hospital completeness of discharge summaries data
Supplementary Figure17. Inclusion and exclusion flowchart
Supplementary Figure18. Architecture of the algorithm used to detect hospitalisations caused by suicide attempts
Supplementary Table5. Dictionary used to detect suicide attempts
Supplementary Table6. Dictionary used to detect risk factors

Model’s residuals

Supplementary Figure1 shows that the overall model of the main article’s Eq.1 fitted well the time series of the monthly number of hospitalised SA. Seasonality was in particular well accounted for. Supplementary Figure2 shows that the residuals did not feature any noticeable time trend indicating that temporal variations were correctly accounted for by the model.

Supplementary Figure1. Detected and modelled monthly numbers of suicide attempts

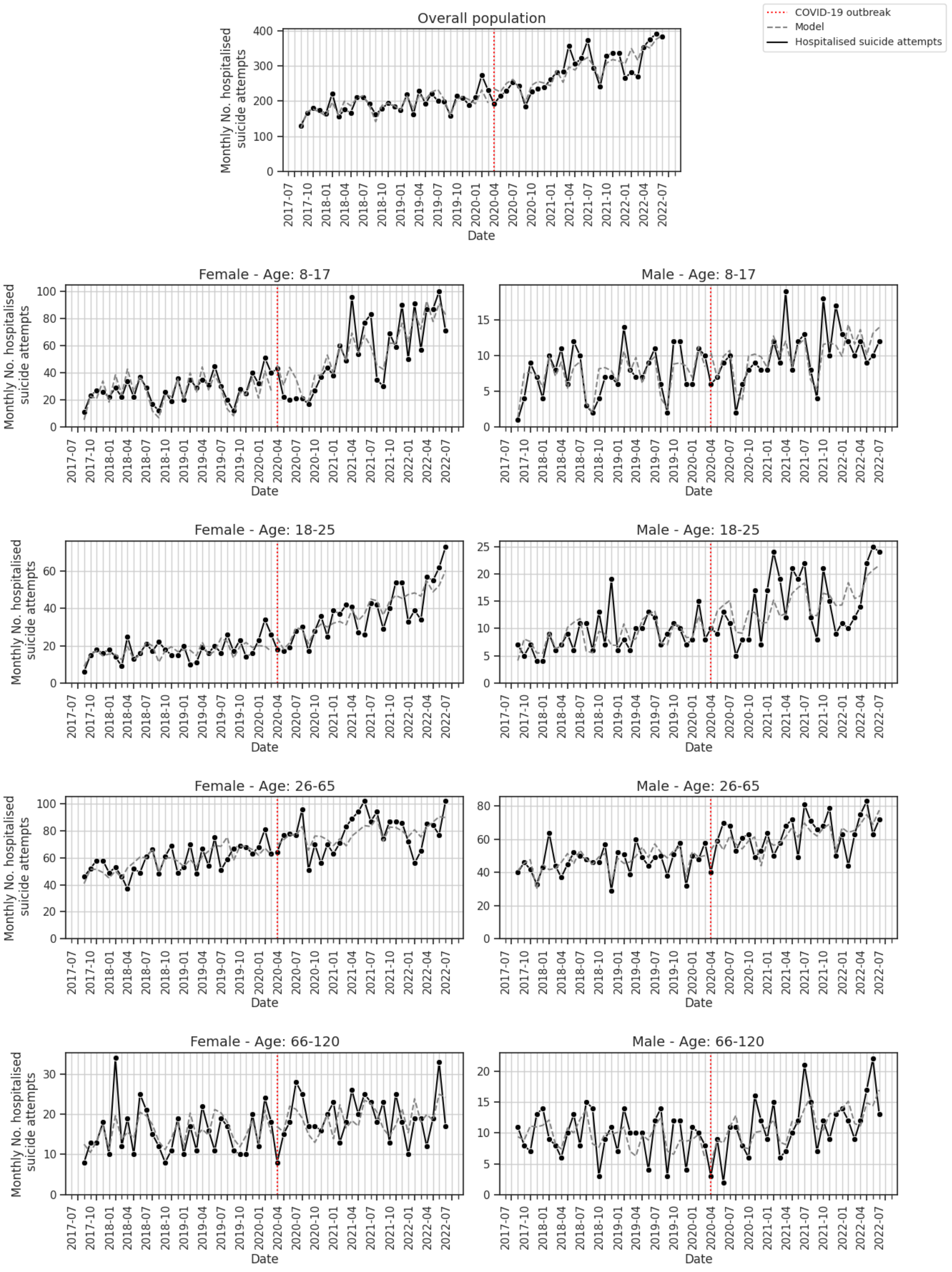

Supplementary Figure2. Model's residuals

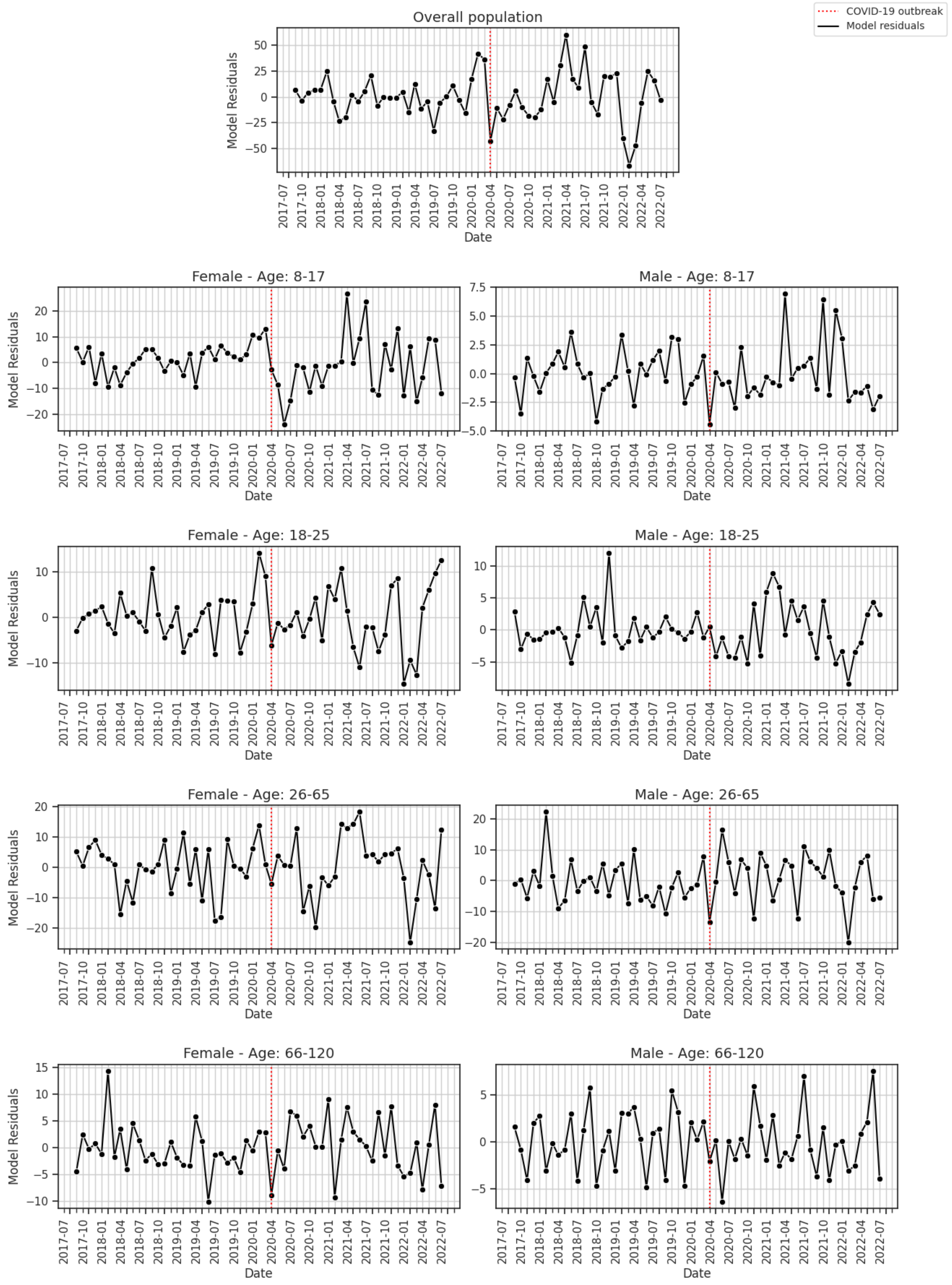

Sensitivity analysis

We assessed the robustness of our conclusions by conducting four sensitivity analyses.

Rule-based algorithm

A first sensitivity analysis consisted in replacing the entity-classification algorithm based on machine learning by a simpler rule-based algorithm (see *Details on the algorithms* section). Although the performances of this algorithm were lower than the machine learning algorithm, the results presented in the main article were robust to this modification.

Supplementary Figure3. Monthly numbers of hospitalisations caused by suicide attempts - rule-based algorithm

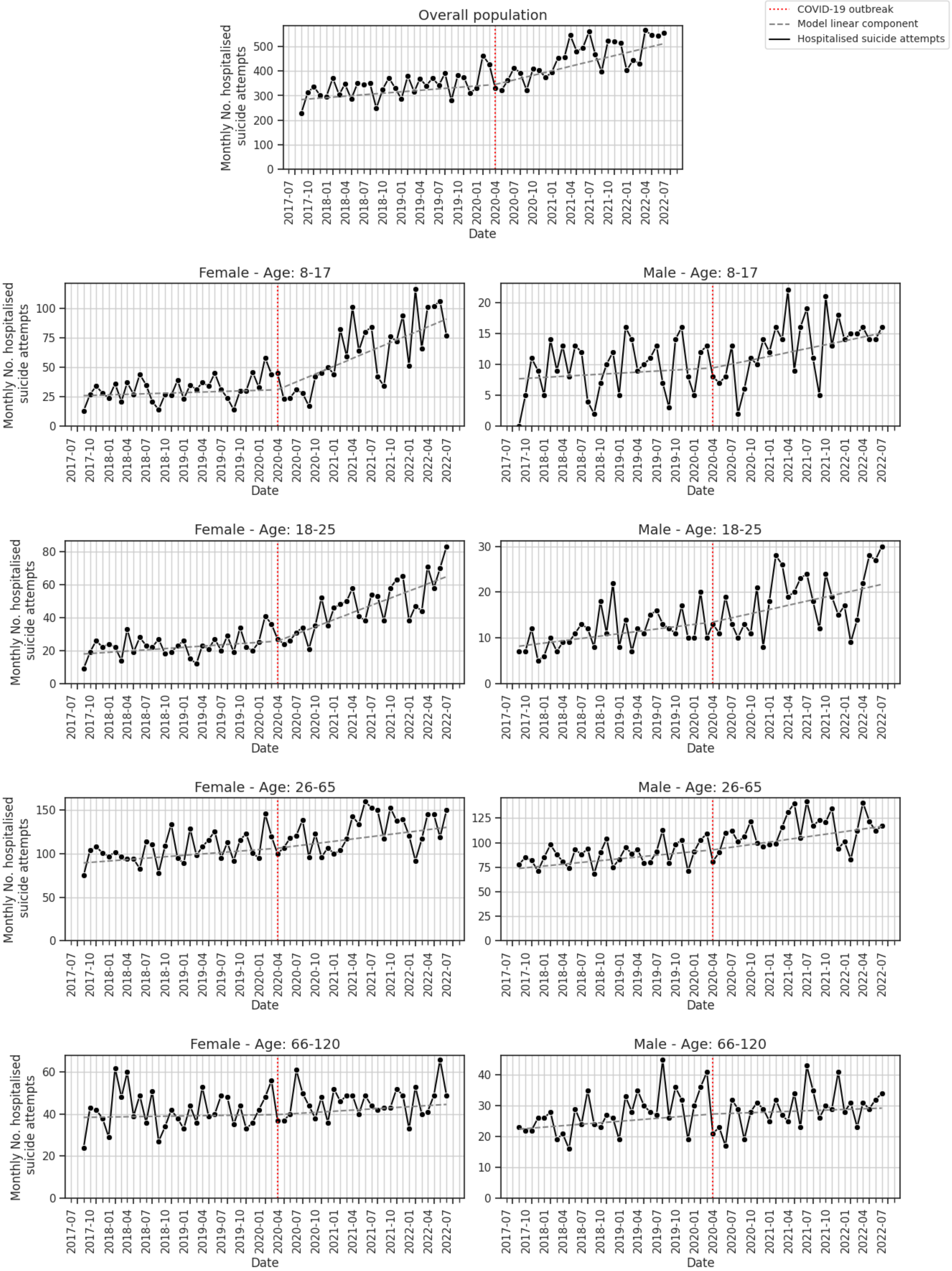

Supplementary Table1. Numbers and modelling of hospitalisations caused by suicide attempts - rule-based algorithm

		**No. hospitalisations caused by suicide attempts (%) - pre-pandemic period**	**No. hospitalisations caused by suicide attempts (%) - post-pandemic period**	**Long term mean α_0 (95%CI)**	**Long term trend α_1 (95%CI)**	**Trend variation after COVID-19 outbreak α_2 (95%CI)**
**Male**	**8-17**	290 (2.8%)	359 (2.8%)	7.7 (5.9–9.5)	0.1 (-0.0–0.1)	0.2 (-0.0–0.3)
	**18-25**	353 (3.4%)	512 (4.0%)	8.2 (5.3–11.0)	0.2 (0.0–0.3)	0.1 (-0.2–0.4)
	**26-65**	2731 (26.1%)	3127 (24.7%)	73.8 (66.0–81.5)	0.6 (0.2–1.0)	0.3 (-0.5–1.1)
	**66-**	856 (8.2%)	807 (6.4%)	22.4 (18.9–25.9)	0.2 (-0.0–0.3)	-0.1 (-0.4–0.3)
	**All Ages**	4230 (40.4%)	4805 (38.0%)	112.0 (101.1–123.0)	1.0 (0.4–1.6)	0.5 (-0.6–1.6)
**Female**	**8-17**	968 (9.2%)	1756 (13.9%)	25.9 (19.4–32.5)	0.2 (-0.2–0.5)	2.1 (1.4–2.7)
	**18-25**	718 (6.9%)	1308 (10.3%)	17.9 (13.1–22.8)	0.3 (-0.0–0.5)	1.2 (0.7–1.7)
	**26-65**	3263 (31.1%)	3505 (27.7%)	89.5 (80.1–99.0)	0.5 (0.1–1.0)	0.3 (-0.6–1.3)
	**66-**	1298 (12.4%)	1280 (10.1%)	38.5 (33.8–43.1)	0.0 (-0.2–0.3)	0.1 (-0.3–0.6)
	**All Ages**	6247 (59.6%)	7849 (62.0%)	171.8 (157.0–186.7)	1.0 (0.2–1.8)	3.7 (2.2–5.2)
**Overall**		10477 (100.0%)	12654 (100.0%)	283.9 (261.2–306.6)	2.0 (0.8–3.2)	4.2 (1.9–6.5)

Claim-based algorithm

In another sensitivity analysis we selected among the total database hospitalisations that had at least one claim code related to self-harm (X60-X84 codes of the International Classification of Diseases, 10th revision, as in previous studies, extracted from the French PMSI, *Programme de Médicalisation des Systèmes d'Information* database).[1] We underline the limits of this approach as codes X62 (self-intoxication by narcotics and hallucinogens) and X65 (self-intoxication by alcohol) may in particular not correspond to our definition of SA. These codes are not restricted to suicide attempts but they are commonly used by clinicians to report them. Another limitation was that claim data was only available for stays in medicine, surgery and obstetrics hospital departments. The periods of claim data's availability in the database is equal to the period of administrative data availability, and we considered consequently the same hospitals as in the main analysis. Considering all the hospitalisation stays that were labelled as SA-caused using claim data, we obtained the following results. The variation of trend for the overall population appeared smaller than the one obtained by the NLP algorithm and no significant variation is observed for young women (aged 18-25) but we still observed a positive variation for girls (aged 8-17).

Supplementary Figure4. Monthly numbers of hospitalisations caused by suicide attempts - claim data

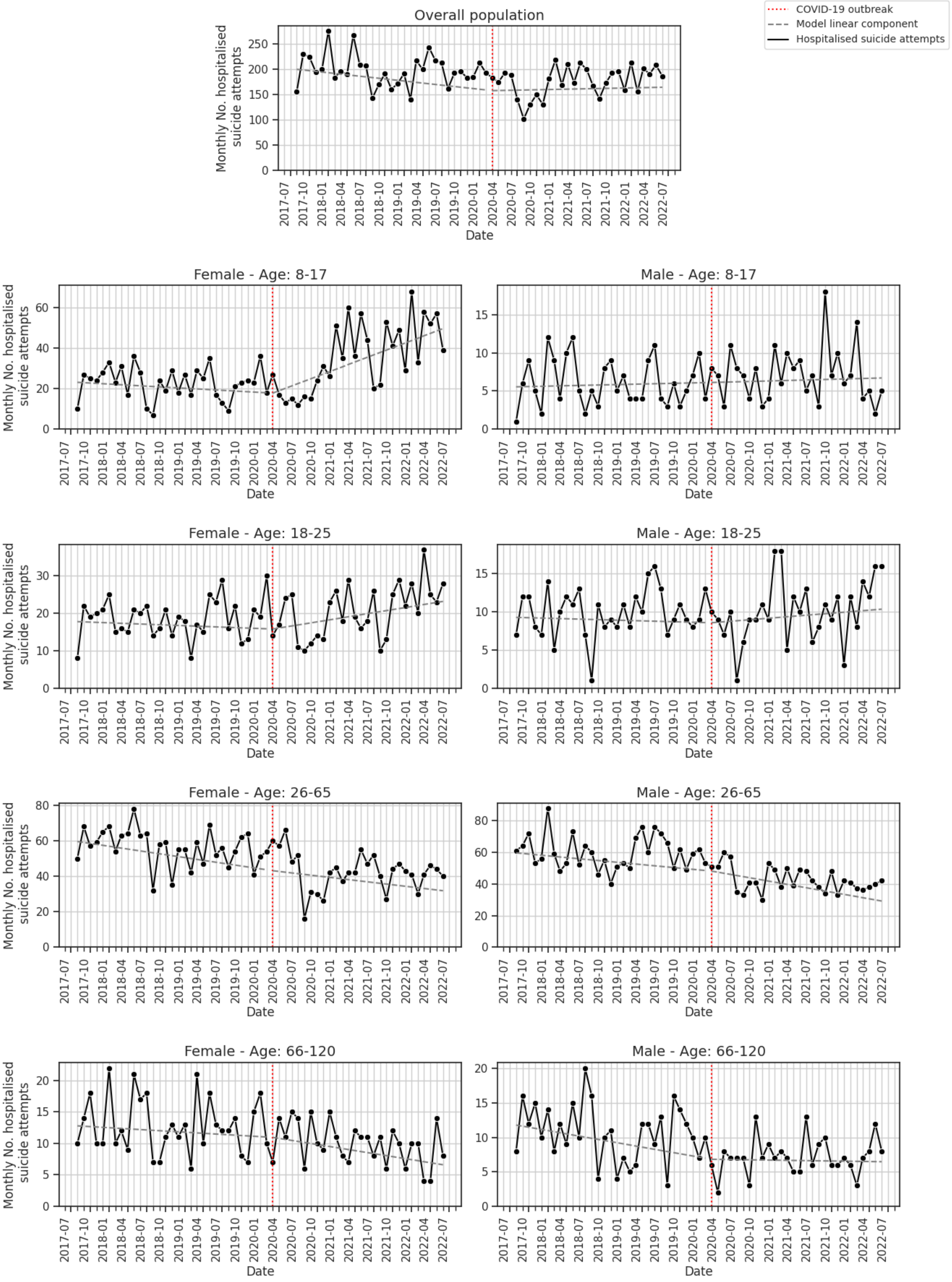

Supplementary Table2. Numbers and modelling of hospitalisations caused by suicide attempts - claim algorithm

		**No. hospitalisations caused by suicide attempts (%) - pre-pandemic period**	**No. hospitalisations caused by suicide attempts (%) - post-pandemic period**	**Long term mean α_0 (95%CI)**	**Long term trend α_1 (95%CI)**	**Trend variation after COVID-19 outbreak α_2 (95%CI)**
**Male**	**8-17**	188 (3.1%)	200 (4.1%)	5.5 (3.4–7.6)	0.0 (-0.1–0.1)	0.0 (-0.2–0.2)
	**18-25**	306 (5.0%)	284 (5.8%)	9.3 (7.4–11.2)	-0.0 (-0.1–0.1)	0.1 (-0.1–0.3)
	**26-65**	1852 (30.4%)	1196 (24.3%)	59.6 (54.2–64.9)	-0.4 (-0.6 - -0.1)	-0.3 (-0.9–0.2)
	**66-**	330 (5.4%)	202 (4.1%)	11.8 (9.6–14.0)	-0.2 (-0.3 - -0.0)	0.1 (-0.1–0.4)
	**All Ages**	2676 (43.9%)	1882 (38.2%)	86.2 (79.0–93.3)	-0.5 (-0.9 - -0.2)	-0.1 (-0.8–0.6)
**Female**	**8-17**	706 (11.6%)	1000 (20.3%)	23.1 (18.8–27.4)	-0.2 (-0.4–0.1)	1.4 (0.9–1.8)
	**18-25**	576 (9.4%)	575 (11.7%)	17.8 (14.5–21.1)	-0.1 (-0.2–0.1)	0.3 (0.0–0.7)
	**26-65**	1743 (28.6%)	1191 (24.2%)	59.5 (54.5–64.6)	-0.5 (-0.8 - -0.3)	0.1 (-0.4–0.6)
	**66-**	397 (6.5%)	279 (5.7%)	12.8 (10.6–15.0)	-0.1 (-0.2–0.1)	-0.1 (-0.3–0.1)
	**All Ages**	3422 (56.1%)	3045 (61.8%)	113.2 (105.1–121.3)	-0.8 (-1.2 - -0.4)	1.7 (0.9–2.5)
**Overall**		6098 (100.0%)	4927 (100.0%)	199.4 (186.5–212.3)	-1.4 (-2.0 - -0.7)	1.6 (0.3–2.9)

Adjusting for potential deployment bias

Another sensitivity analysis consisted in adjusting for a potential bias induced by the temporally unequal availability of discharge summaries (see Supplementary Figure17). For each hospital and each month, the number of detected SA-caused hospitalisations was divided by the proportion of hospitalisations having at least one discharge summary available in the research database (i.e., not restricted to SA-caused stays). Assuming that discharge summaries were missing completely at random (MCAR assumption), dividing by data completeness indeed provides an estimate of the true number of SA-caused hospitalisations. Modifying this single aspect, we obtained the following results that were coherent with the main analysis.

Supplementary Figure5. Monthly numbers of hospitalisations caused by suicide attempts - completeness adjusted

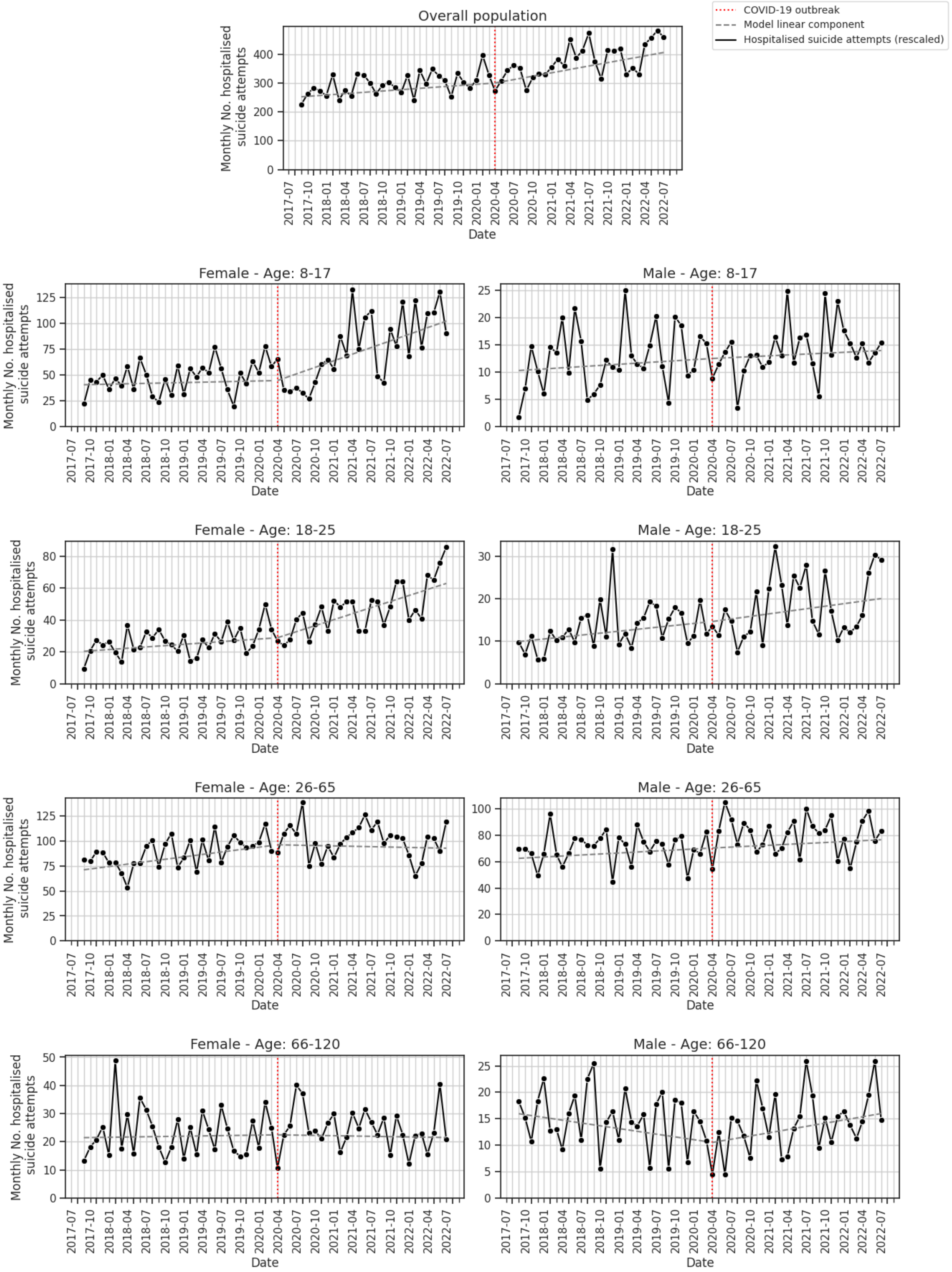

Supplementary Table3. Numbers and modelling of hospitalisations caused by suicide attempts - completeness adjusted

		**No. hospitalisations caused by suicide attempts (%) - pre-pandemic period**	**No. hospitalisations caused by suicide attempts (%) - post-pandemic period**	**Long term mean α_0 (95%CI)**	**Long term trend α_1 (95%CI)**	**Trend variation after COVID-19 outbreak α_2 (95%CI)**
**Male**	**8-17**	388 (4.2%)	390 (3.7%)	10.3 (7.7–12.9)	0.1 (-0.1–0.2)	-0.0 (-0.3–0.2)
	**18-25**	409 (4.5%)	507 (4.8%)	9.9 (6.3–13.5)	0.2 (-0.0–0.3)	0.0 (-0.3–0.4)
	**26-65**	2173 (23.7%)	2239 (21.4%)	62.4 (55.3–69.6)	0.3 (-0.1–0.6)	-0.0 (-0.7–0.7)
	**66-**	460 (5.0%)	397 (3.8%)	15.9 (12.8–19.1)	-0.2 (-0.3 - -0.0)	0.4 (0.1–0.7)
	**All Ages**	3430 (37.4%)	3533 (33.7%)	98.6 (89.5–107.7)	0.3 (-0.2–0.8)	0.4 (-0.5–1.3)
**Female**	**8-17**	1462 (16.0%)	2128 (20.3%)	40.6 (32.1–49.0)	0.1 (-0.3–0.6)	2.0 (1.1–2.9)
	**18-25**	821 (9.0%)	1319 (12.6%)	20.4 (14.8–25.9)	0.3 (-0.0–0.6)	1.0 (0.4–1.5)
	**26-65**	2740 (29.9%)	2820 (26.9%)	71.2 (62.7–79.7)	0.8 (0.4–1.2)	-0.9 (-1.8 - -0.1)
	**66-**	710 (7.7%)	686 (6.5%)	21.4 (17.0–25.9)	0.0 (-0.2–0.3)	-0.1 (-0.5–0.4)
	**All Ages**	5734 (62.6%)	6953 (66.3%)	153.6 (138.5–168.6)	1.2 (0.5–2.0)	2.0 (0.5–3.5)
**Overall**		9164 (100.0%)	10486 (100.0%)	252.2 (233.4–270.9)	1.5 (0.6–2.5)	2.4 (0.5–4.3)

Per-hospital subgroup analysis

Another sensitivity analysis consisted in conducting per-hospital subgroup analyses. We considered successively each one of the 15 hospitals (see Supplementary Table4 for abbreviations), and reproduced the same analysis. We focused on the trend variations after COVID-19 for the overall population and for the 8-17 female population.

- *Main (hybrid) algorithm for stay-classification*

Supplementary Figure6. Per-hospital forest plot of trend variations - overall population

Hospital	Hospital type	No. hospitalised suicide attempts
ABC	Adult & Paediatric	1301
APR	Adult & Paediatric	1812
BCH	Adult	549
BCT	Adult & Paediatric	1014
HMN	Adult	1109
JVR	Adult & Paediatric	511
LMR	Adult & Paediatric	973
LRB	Adult	1412
NCK	Adult & Paediatric	828
PBR	Adult	319
PSL	Adult	1134
SAT	Adult	1538
SLS	Adult	412
TNN	Adult	836
TRS	Paediatric	275

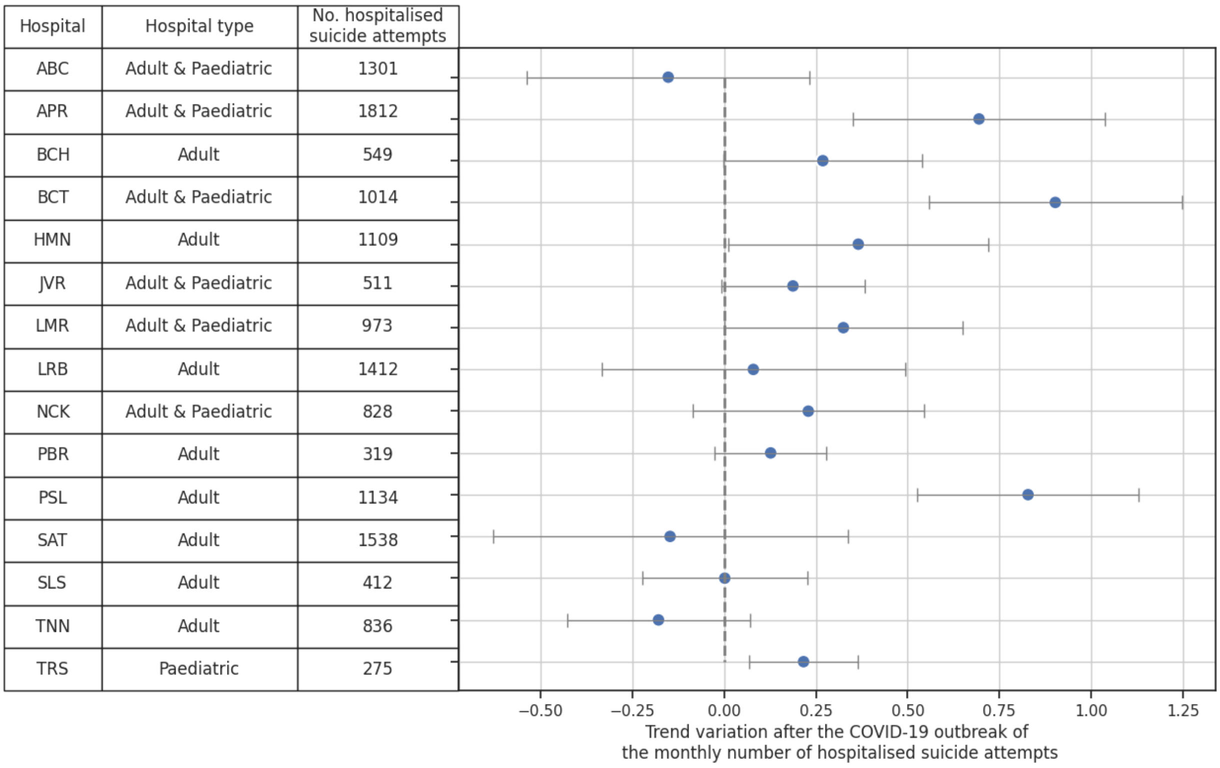

Supplementary Figure7. Per-hospital forest plot of trend variations - girls

Hospital	Hospital type	No. hospitalised suicide attempts
ABC	Adult & Paediatric	311
APR	Adult & Paediatric	314
BCH	Adult	21
BCT	Adult & Paediatric	301
HMN	Adult	28
JVR	Adult & Paediatric	164
LMR	Adult & Paediatric	269
LRB	Adult	48
NCK	Adult & Paediatric	520
PBR	Adult	17
PSL	Adult	84
SAT	Adult	49
SLS	Adult	14
TNN	Adult	36
TRS	Paediatric	224

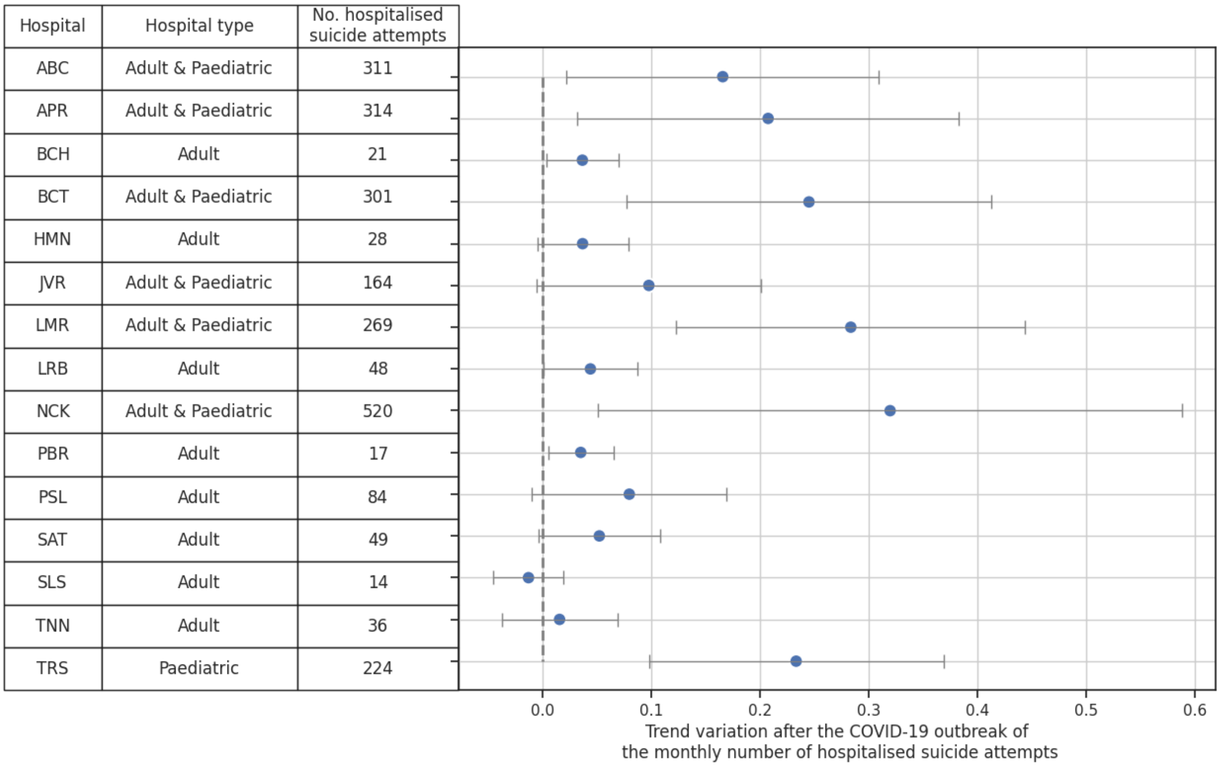

- *Rule-based algorithm for stay-classification*

Supplementary Figure8. Per-hospital forest plot of trend variations - overall population, rule-based algorithm

Hospital	Hospital type	No. hospitalised suicide attempts
ABC	Adult & Paediatric	1667
APR	Adult & Paediatric	2294
BCH	Adult	1258
BCT	Adult & Paediatric	2216
HMN	Adult	2083
JVR	Adult & Paediatric	654
LMR	Adult & Paediatric	1319
LRB	Adult	2492
NCK	Adult & Paediatric	944
PBR	Adult	724
PSL	Adult	2790
SAT	Adult	2416
SLS	Adult	622
TNN	Adult	1330
TRS	Paediatric	322

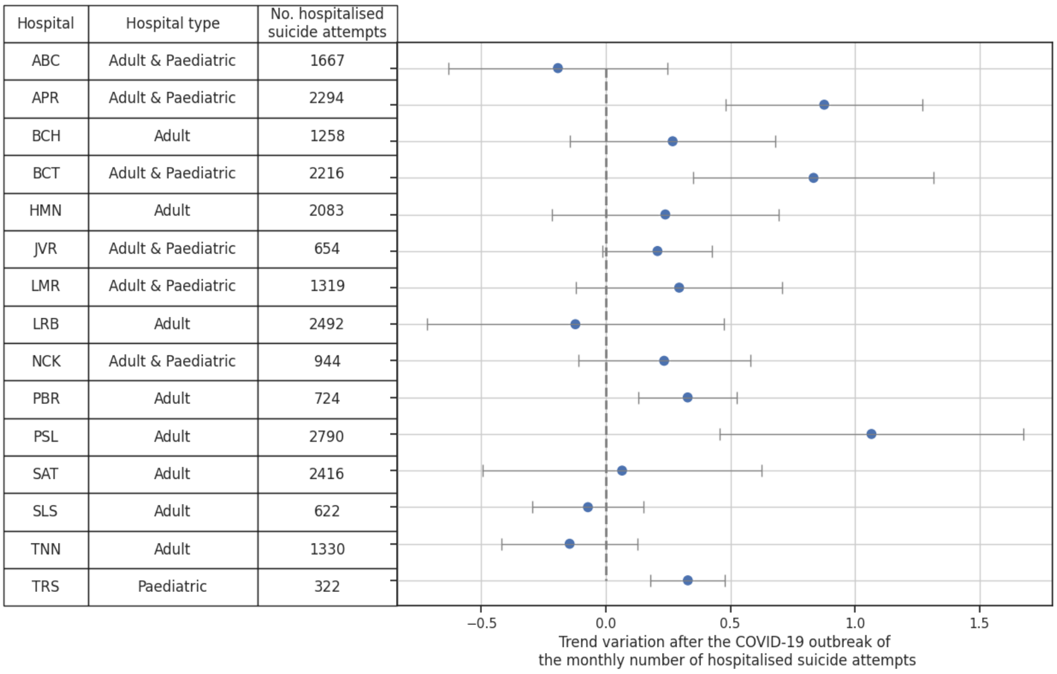

Supplementary Figure9. Per-hospital forest plot of trend variations - girls, rule-based algorithm

Hospital	Hospital type	No. hospitalised suicide attempts
ABC	Adult & Paediatric	351
APR	Adult & Paediatric	341
BCH	Adult	25
BCT	Adult & Paediatric	356
HMN	Adult	30
JVR	Adult & Paediatric	198
LMR	Adult & Paediatric	327
LRB	Adult	51
NCK	Adult & Paediatric	555
PBR	Adult	19
PSL	Adult	118
SAT	Adult	54
SLS	Adult	14
TNN	Adult	38
TRS	Paediatric	247

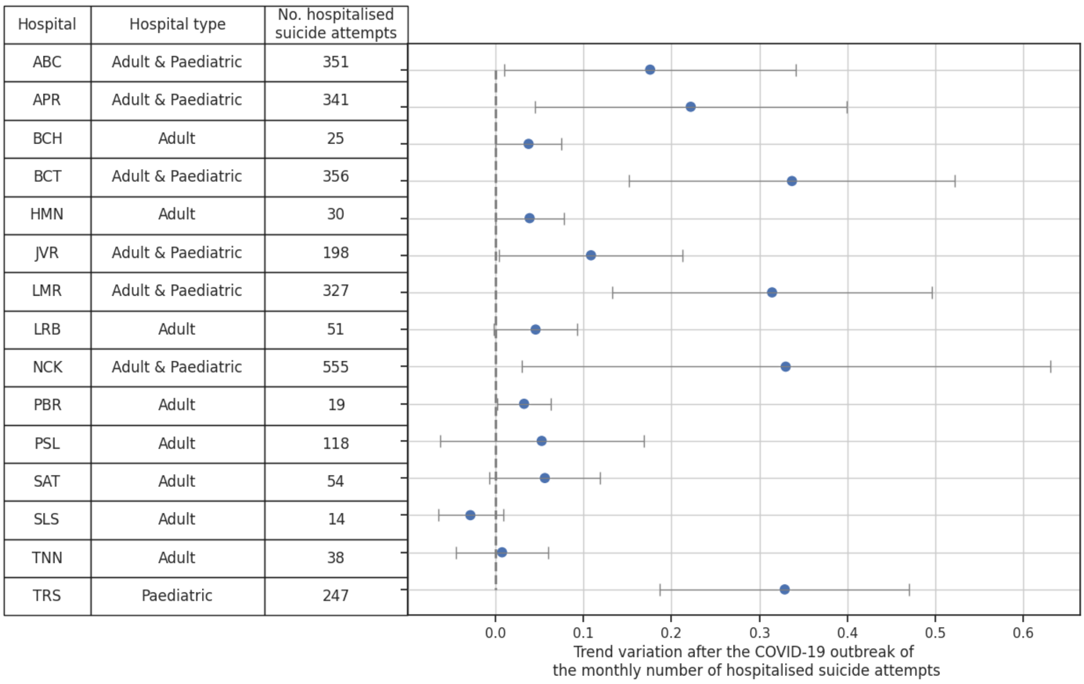

- *Completeness-adjusted monthly numbers of SA*

Supplementary Figure10. Per-hospital forest plot of trend variations - overall population, completeness-adjusted

Hospital	Hospital type	No. hospitalised suicide attempts
ABC	Adult & Paediatric	2076
APR	Adult & Paediatric	2076
BCH	Adult	824
BCT	Adult & Paediatric	1381
HMN	Adult	1300
JVR	Adult & Paediatric	1099
LMR	Adult & Paediatric	1908
LRB	Adult	1926
NCK	Adult & Paediatric	1234
PBR	Adult	334
PSL	Adult	1495
SAT	Adult	1730
SLS	Adult	603
TNN	Adult	1234
TRS	Paediatric	432

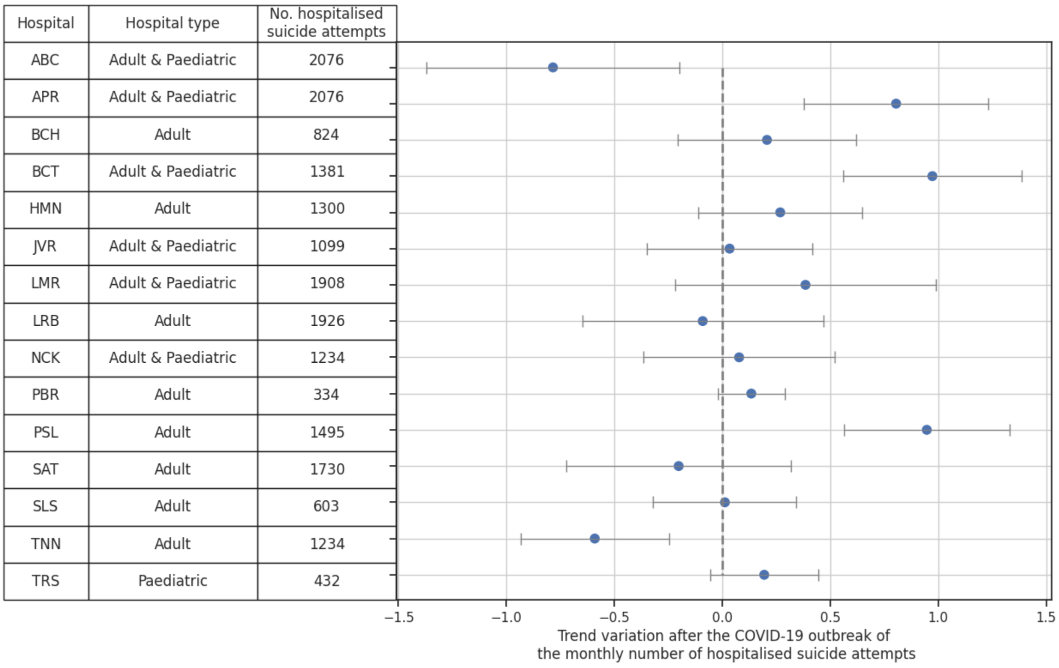

Supplementary Figure11. Per-hospital forest plot of trend variations - girls, completeness-adjusted

Hospital	Hospital type	No. hospitalised suicide attempts
ABC	Adult & Paediatric	488
APR	Adult & Paediatric	354
BCH	Adult	30
BCT	Adult & Paediatric	405
HMN	Adult	31
JVR	Adult & Paediatric	343
LMR	Adult & Paediatric	514
LRB	Adult	65
NCK	Adult & Paediatric	758
PBR	Adult	18
PSL	Adult	112
SAT	Adult	54
SLS	Adult	21
TNN	Adult	50
TRS	Paediatric	347

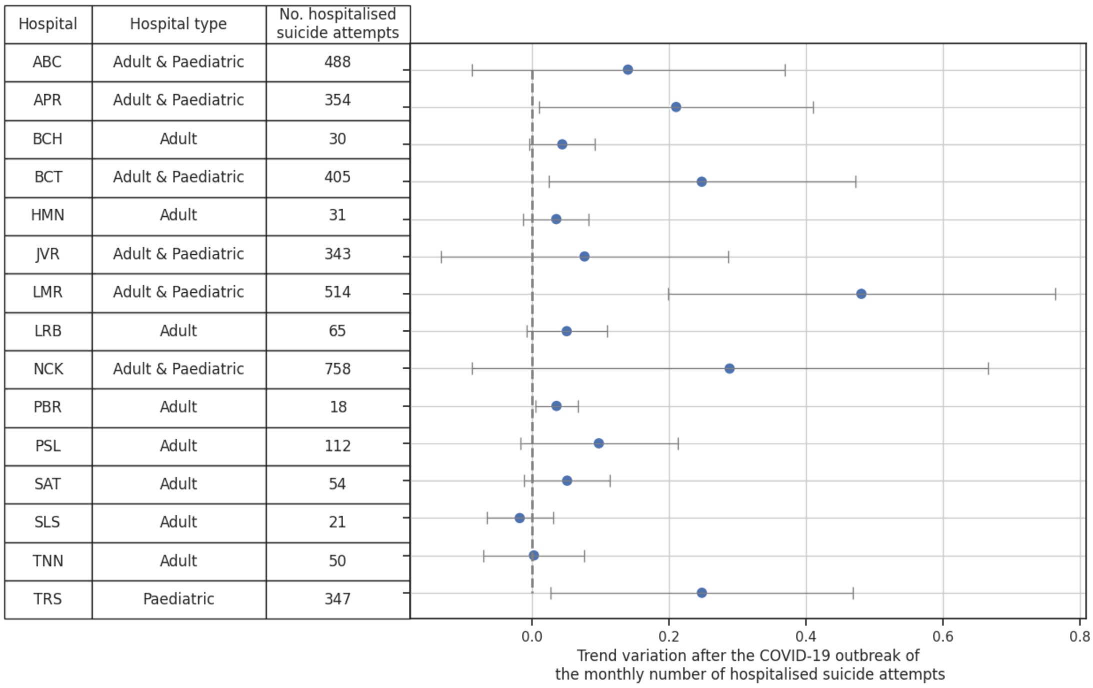

- *Claim-based algorithm*

Supplementary Figure12. Per-hospital forest plot of trend variations - overall population, claim-based algorithm

Hospital	Hospital type	No. hospitalised suicide attempts
ABC	Adult & Paediatric	1670
APR	Adult & Paediatric	877
BCH	Adult	1001
BCT	Adult & Paediatric	1050
HMN	Adult	1023
JVR	Adult & Paediatric	377
LMR	Adult & Paediatric	494
LRB	Adult	1009
NCK	Adult & Paediatric	501
PBR	Adult	74
PSL	Adult	741
SAT	Adult	1106
SLS	Adult	319
TNN	Adult	509
TRS	Paediatric	274

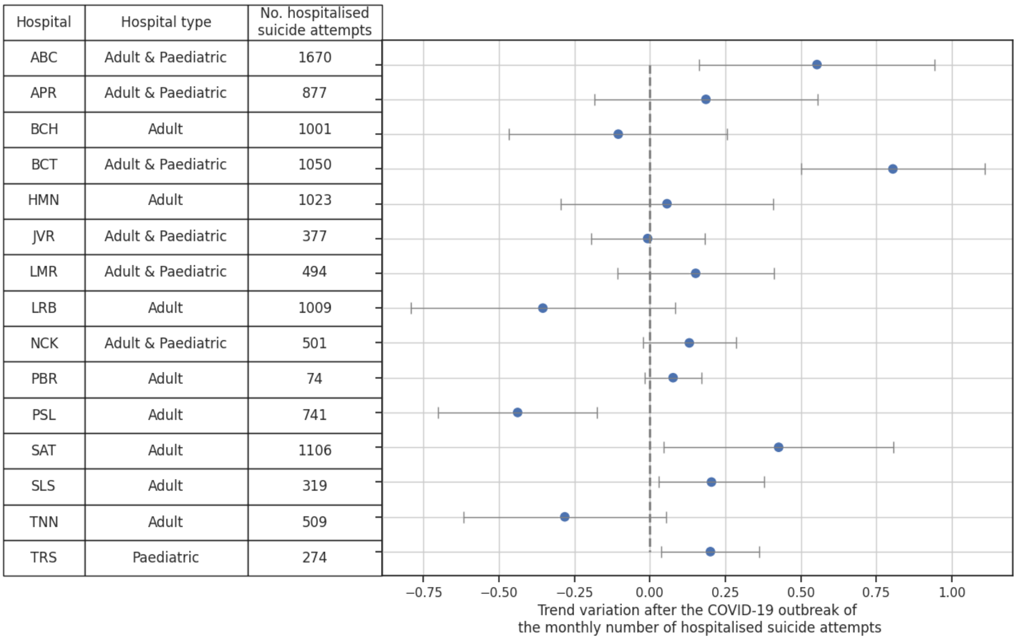

Supplementary Figure13. Per-hospital forest plot of trend variations - girls, claim-based algorithm

Hospital	Hospital type	No. hospitalised suicide attempts
ABC	Adult & Paediatric	285
APR	Adult & Paediatric	198
BCH	Adult	38
BCT	Adult & Paediatric	287
HMN	Adult	36
JVR	Adult & Paediatric	145
LMR	Adult & Paediatric	125
LRB	Adult	30
NCK	Adult & Paediatric	260
PBR	Adult	11
PSL	Adult	17
SAT	Adult	28
SLS	Adult	9
TNN	Adult	23
TRS	Paediatric	214

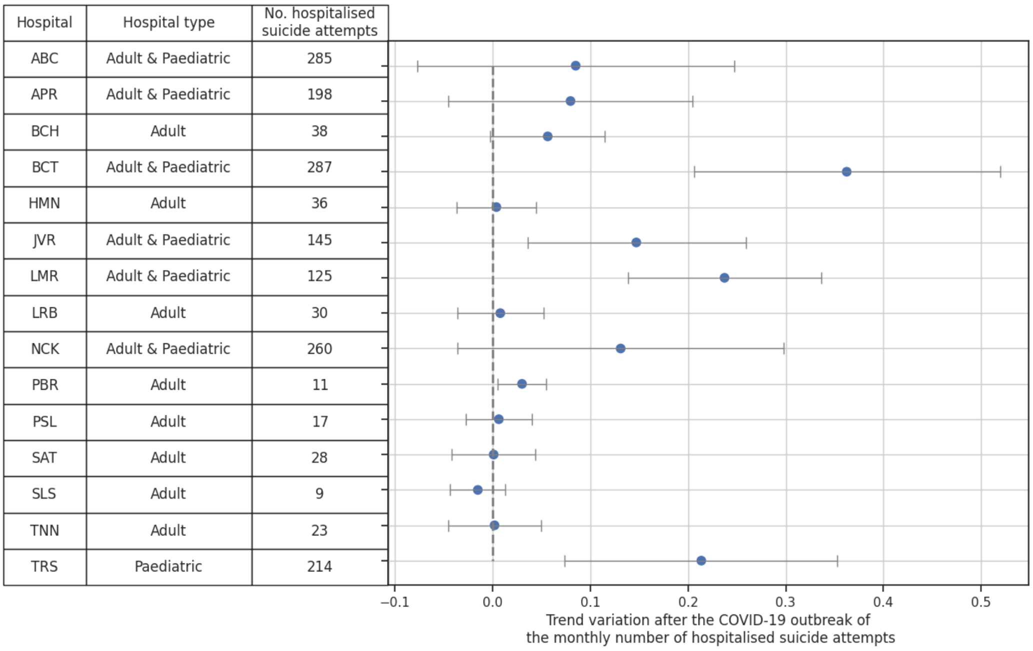

Modalities of suicide attempt

Supplementary Figure3 shows the variation with time of the proportion of each modality of suicide attempt. We observed a stable proportion of each modality with respect to time, and a larger amount of intentional drug intoxications for females compared to males. If many different positive mentions of modalities were detected in the discharge summary of a SA-caused stay, we weighted each modality proportionally to the number of mentions (i.e., if a stay mentioned 3 times drug intoxications and 2 times defenestration, we counted 3/(3+2) drug intoxication and 2/(3+2) defenestration in the aggregate result shown in Supplementary Figure3). Generic mentions (e.g., "suicide attempt") were not counted if other positive modalities were found. Otherwise, if there were only generic mentions, the stay was labelled as *"Unknown & other forms"*.

Supplementary Figure14. Monthly proportions of each modality of suicide attempt

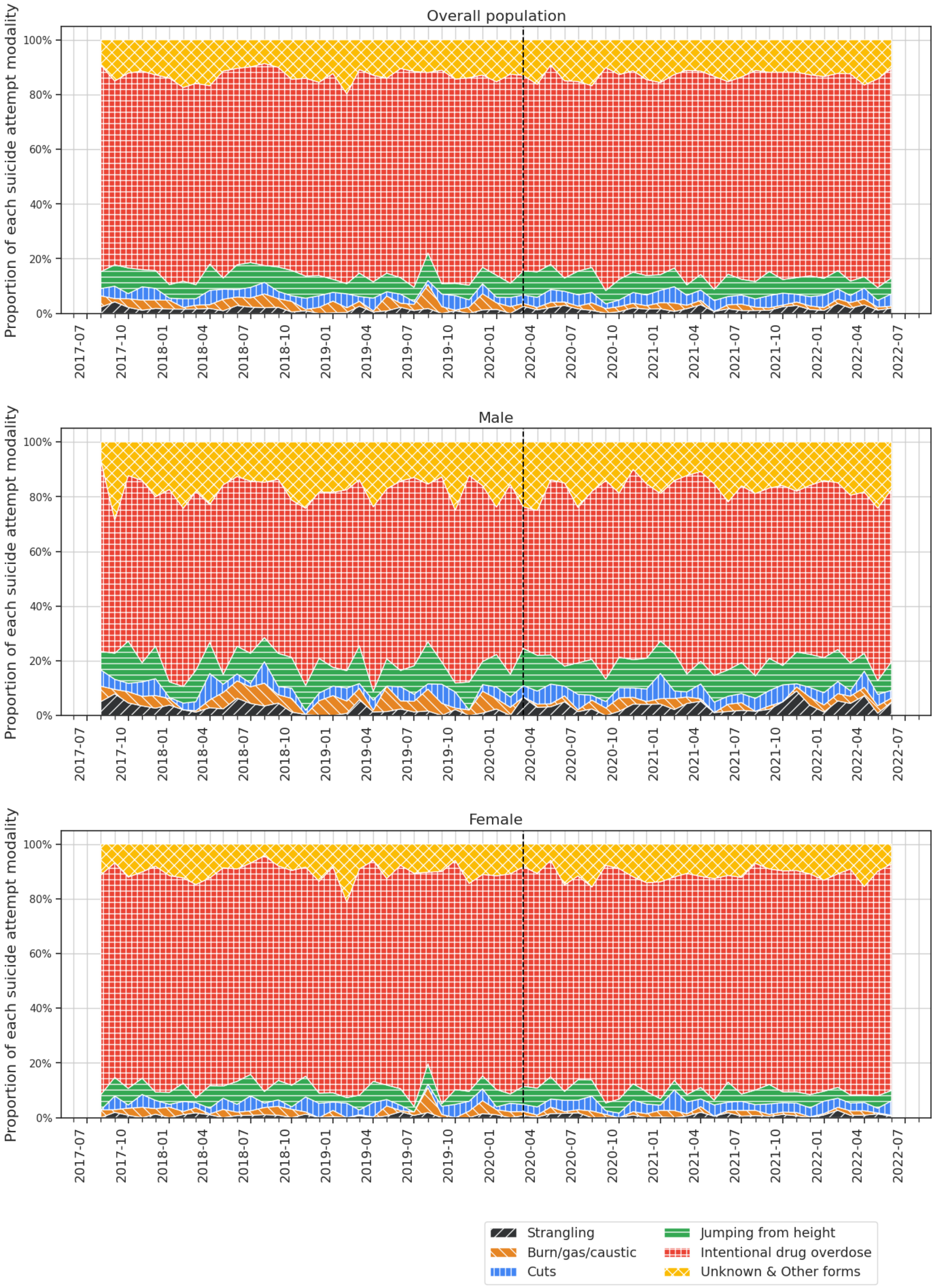

Severity analysis

To study the severity of SA-caused stays accounting for the potential bias induced by censoring of non-terminated stays, we considered length of stay and death during stay as censored data and compared pre- and post-pandemic groups using a logrank tests and plotting the associated Kaplan-Meier curves.

Supplementary Figure15. Kaplan-Meier curves relative to stay duration (A) and death during stay (B)

A)

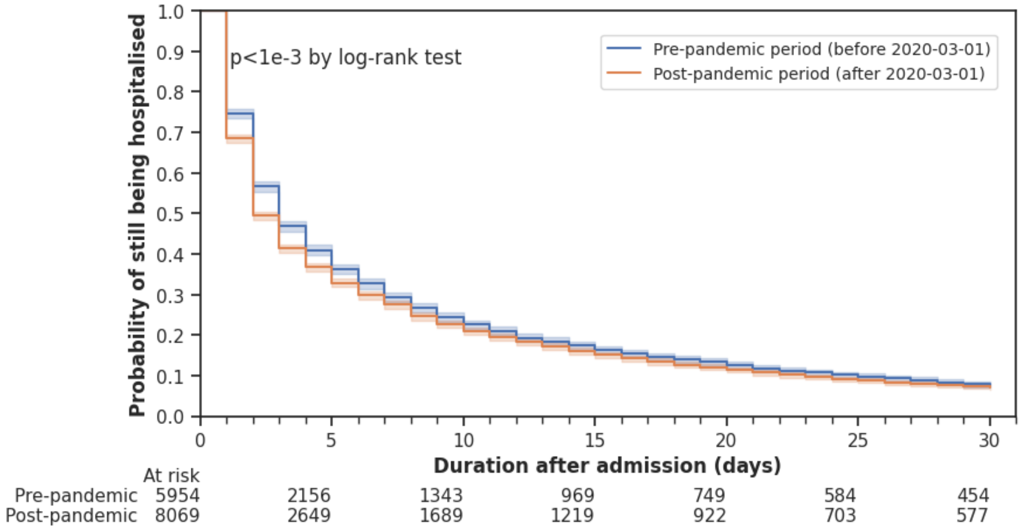

B)

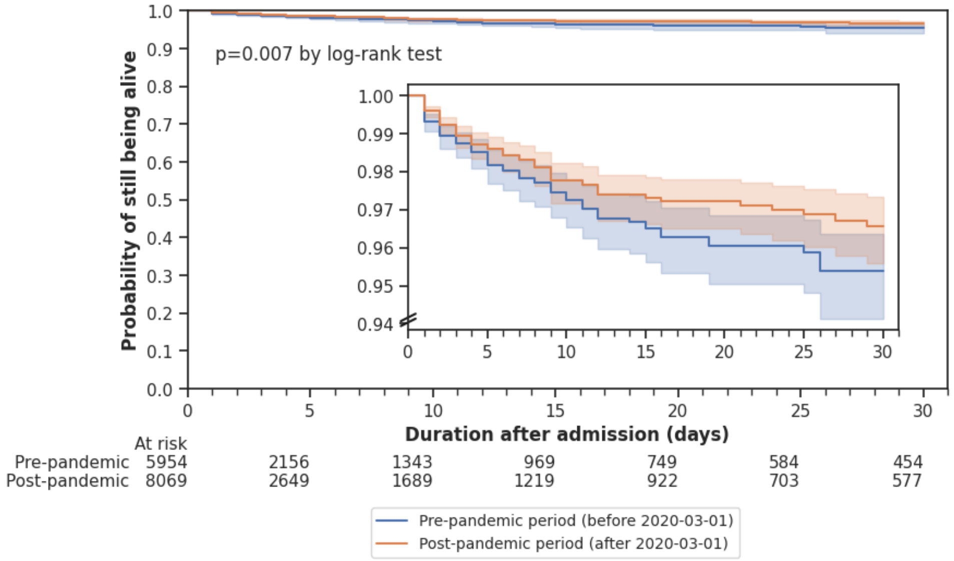

Details on cohort creation

The source population was restricted to 15 of the 38 AP-HP's hospitals in order to limit data completeness issues. Indeed, only the main and most recent softwares of the clinical information system have seen their data integrated in the database. The deployment of the main electronic health record (EHR) software, ORBIS Dedalus Healthcare, is in particular an ongoing process that started in 2012 and is not yet achieved. Depending on the state of the deployment at a given date in a given medical unit, data may or may not be available in the database for secondary use. In this study we therefore considered only 15 of the 38 AP-HP's hospitals for which the deployment of the EHR was considered as advanced at the beginning of the study (Supplementary Table4). Supplementary Figure16 shows for each one of the 15 included hospitals the proportion of all the hospital stays (i.e., not specific to SA) that have at least one discharge summary available in the research database. One observed a slow variation with time of data completeness that depended on the hospital. A dedicated sensitivity analysis was therefore conducted in order to test the robustness of our study's result with respect to this issue (see Supplementary Figure15 and Supplementary Table2).

Supplementary Table4. Hospitals of the Greater Paris University Hospitals considered in this study

**Trigram**	**Hospital Name**	**Number of beds**	**Set**	**Adult/Paediatric**
**ABC**	ANTOINE BECLERE	420	Training	Adult & Paediatric
**APR**	AMBROISE PARE	360	Training	Adult & Paediatric
**BCH**	BICHAT	780	Training	Adult
**BCT**	BICÊTRE	840	Validation	Adult & Paediatric
**HMN**	HENRI MONDOR	830	Validation	Adult
**JVR**	JEAN VERDIER	180	Validation	Adult & Paediatric
**LMR**	LOUIS MOURIER	310	Training	Adult & Paediatric
**LRB**	LARIBOISIERE	600	Training	Adult
**NCK**	NECKER	580	Training	Adult & Paediatric
**PBR**	PAUL BROUSSE	140	Training	Adult
**PSL**	PITIE SALPETRIERE	1410	Training	Adult
**SAT**	SAINT ANTOINE	620	Training	Adult
**SLS**	SAINT LOUIS	530	Training	Adult
**TNN**	TENON	460	Validation	Adult
**TRS**	TROUSSEAU	310	Validation	Paediatric

Supplementary Figure16. Per-hospital completeness of discharge summaries data

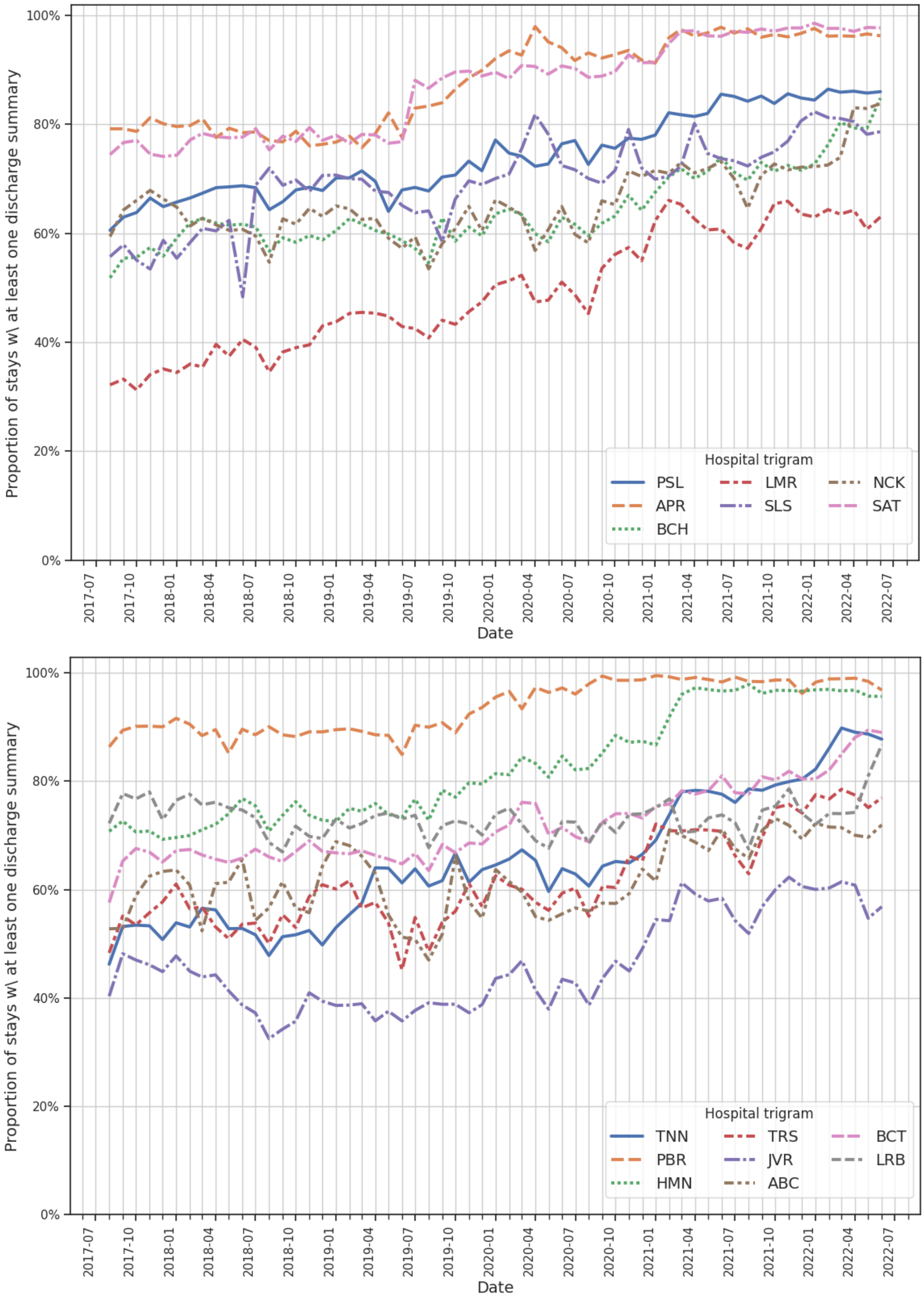

The clinical data warehouse of the Greater Paris University Hospitals features an architecture that imposes a pre-selection of patients using a dedicated query engine (Informatics for Integrating Biology & the Bedside).[2] Once a subset of patients has been selected by authorised data owners, data relative to these patients is made available to investigators in an analysis environment where Python computer code can be executed.

In this study the detection of stays caused by suicide attempts (SA) was consequently decomposed in two steps. A first keyword-based step was designed to be extremely sensitive at the cost of a low specificity (i.e., screening stage) and a second step leveraged an advanced

stay-classification algorithm to discard false positive detections (Supplementary Figure17). The keyword selection step could not rely on word stemming as this functionality was not available in the query engine. Nevertheless, at this step we selected a patient subset and not their clinical documents individually. Consequently, if at least one SA keyword was detected in any clinical document of a patient, she was included. The redundancy of SA keywords in records of patients having truly experienced SA consequently balanced the intrinsic limitations of the pre-selection query engine by ensuring a high sensitivity.[3]

Supplementary Figure17. Inclusion and exclusion flowchart

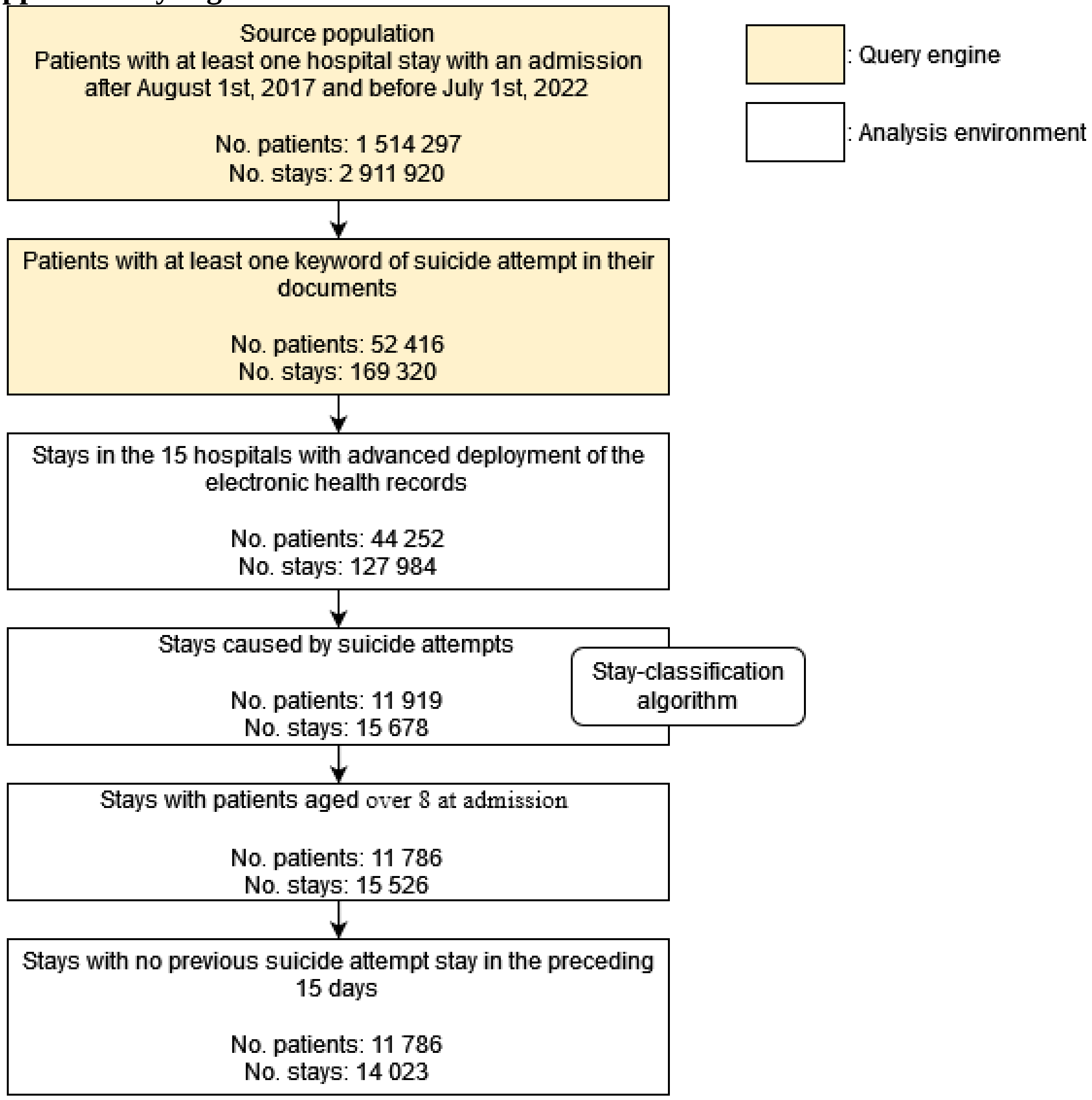

The initial selection steps were executed using a dedicated query engine to select patients among the total database (screening, yellow). The final selection steps relied on the execution of advanced algorithms and filters developed for this study in an analysis environment (white).

Details on the algorithms

Description

Stay-classification algorithm

The stay-classification algorithm used to detect hospitalisations caused by suicide attempts was composed of multiple steps (Supplementary Figure18). Documents were first filtered to include only those that were relative to a hospitalisation (i.e., administrative records described as *"Hospitalisation Complète"* in the EHR) and starting between August 1st, 2017 and June 31st, 2022. Then we selected only discharge summaries or documents which types were not available (recorded as "CRH-Chir", "CRH-Hospi", "CRH-J", "CRH-Neurol" ,"CRH-Pedia" ,"CRH-S" ,"LT-Sor" or "CR:Inconnu" in the EHR software). With this filter we avoided searching in documents of other types that could have led to false positive detections (e.g., pathology report containing the "TS" abbreviation standing for "tissu sain" -healthy tissue- and not "tentative de suicide" -suicide attempt-). Then, only one discharge summary was selected for each visit. This selection is done by prioritising first the documents which type was known (i.e. not typed as "*CR:Inconnu"*) and then among the documents kept, the most recent ones. Then a dictionary of SA terms was used to preselect only the hospitalisations with a discharge summary that included at least one of the dictionary's terms. Each discharge summary was then passed to a document-classification algorithm.

The dictionary of suicide attempt terms was expressed first as simple keywords to be executed in the query engine and second as regular expressions used in the stay-classification algorithm. Regular expressions are syntactically richer than simple keywords, and they can consequently account for some typographic errors or discard some false positive detections whose cause is identified. SA or risk factors (RF) may be expressed by many different keywords or regular expressions that can themselves be grouped in modalities. Supplementary Table5 and Supplementary Table6 show, respectively for the SA detection algorithm and for the RF detection algorithms, the SA modalities/RF and their associated keywords and regular expressions.

Supplementary Figure18. Architecture of the algorithms used to detect hospitalisations caused by suicide attempts

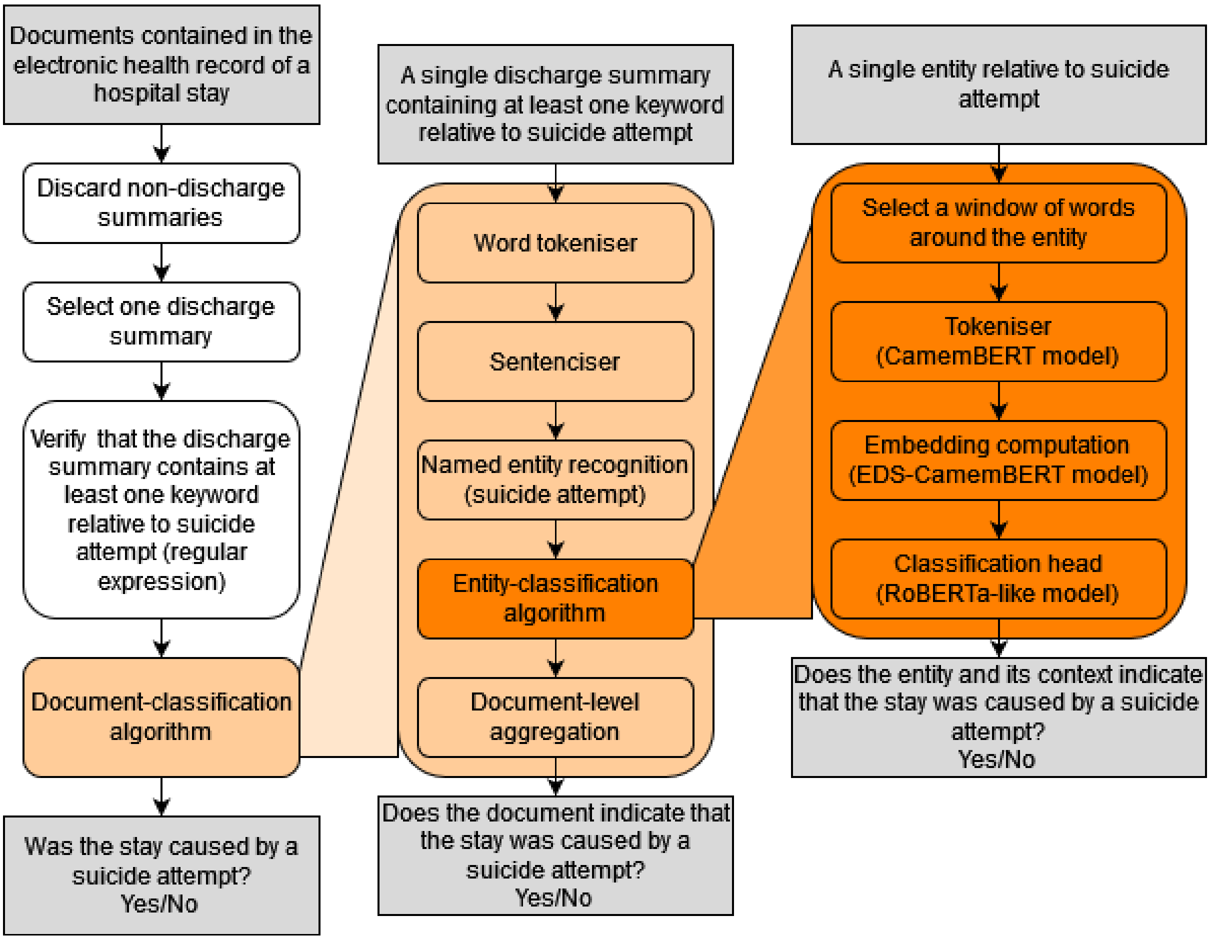

Supplementary Table5. Dictionary used to detect suicide attempts

<table>
<tr><th>Suicide attempt modality (English)</th><th>Suicide attempt modality (French)</th><th>Keywords used in the query engine (French)</th><th>Regular expressions used in the stay-classification algorithm (French)</th></tr>
<tr><td rowspan="11">Jumping from height</td><td rowspan="10">Défenestration</td><td>(defenestration)</td><td rowspan="10">(?i)tentative[s]?\s+de\s+d[ée]fenestration
(?i)(?<!id[ée]e\sde\s)d[ée]fenestration(?!\saccidentelle)
(?i)d[ée]fenestration\s+volontaire
(?i)d[ée]fenestration\s+intentionnelle</td></tr>
<tr><td>(défenestration)</td></tr>
<tr><td>(defenestration & intentionnelle)</td></tr>
<tr><td>(défenestration & intentionnelle)</td></tr>
<tr><td>(defenestration & volontaire)</td></tr>
<tr><td>(défenestration & volontaire)</td></tr>
<tr><td>(tentatives & de & defenestration)</td></tr>
<tr><td>(tentatives & de & défenestration)</td></tr>
<tr><td>(tentative & de & defenestration)</td></tr>
<tr><td>(tentative & de & défenestration)</td></tr>
<tr><td>Jet d’un pont</td><td>-</td><td>(?i)jet.r?\sd.un\spont</td></tr>
<tr><td rowspan="4">Intentional drug overdose</td><td rowspan="4">Intoxication medicamenteuse volontaire</td><td>(i.m.v)</td><td rowspan="4">(?i)(intoxication|ingestion)\s+m[ée]dicamenteuse\s+volontaire
(?i)\b(i\.?m\.?v\.?)\b
(?i)(intoxication|ingestion)\s*([a-zA-Z0-9_éàèôê\-]+\s*){0,3}\s*volontaire</td></tr>
<tr><td>(i.m.v.)</td></tr>
<tr><td>(i.mv)</td></tr>
<tr><td>(i.mv.)</td></tr>
</table>

<table>
<tr><td rowspan="6"></td><td rowspan="6"></td><td>(im.v)</td><td rowspan="6"></td></tr>
<tr><td>(im.v.)</td></tr>
<tr><td>(imv)</td></tr>
<tr><td>(imv.)</td></tr>
<tr><td>(intoxication & medicamenteuse & volontaire)</td></tr>
<tr><td>(intoxication & médicamenteuse & volontaire)</td></tr>
<tr><td>Strangling</td><td>Pendaison</td><td>(pendaison)</td><td>(?i)pendaison</td></tr>
<tr><td rowspan="2">Cuts</td><td rowspan="2">Phlébotomie</td><td>(phlebotomie)</td><td rowspan="2">(?i)phl[ée]botomie</td></tr>
<tr><td>(phlébotomie)</td></tr>
<tr><td>Burn / Gas / Caustic</td><td>Produit caustique</td><td>-</td><td>(?i)ingestion\sde\s(produit\s)?caustique</td></tr>
<tr><td rowspan="8">Unknown & Other forms</td><td rowspan="6">Tentative de suicide</td><td>(t.s)</td><td rowspan="4">\b(?<!\.)(?<!Voie\s\d\s\:\s)(?<!Voie\sd.abord\s\:\s)(?<!surface\s)(?<!d[ée]sorientation\s)(?<!abord\s)(?<!ECG\s:\s)(?<!volume\s)(?<!\d\s[mc]m\sde\sla\s)(?<!\d[mc]m\sde\sla\s)(?<!au\scontact\sde\sla\s)T\.?S\.?(?![\.A-Za-z])(?!\sapyr[eé]tique)(?!.+TRANSSEPTAL)(?!.+T[34])(?!.+en\sr.gression)\b
(?<!\.)T\.S\.(?![A-Za-z])
\b(?<!.)TS\.\B
TS\s+med\s+polymedicamenteuse
TS\s+(poly)?([\s-])?m[ée]dicamenteuse</td></tr>
<tr><td>(t.s.)</td></tr>
<tr><td>(ts)</td></tr>
<tr><td>(ts.)</td></tr>
<tr><td>(tentative & de & suicide)</td><td rowspan="2">(?i)tentative[s]?\s+de\s+sui?cide
(?i)tent[ée]\s+de\s+((se\s+(suicider|tuer))|(mettre\s+fin\s+[àa]\s+((ses\s+jours?)|(sa\s+vie))))</td></tr>
<tr><td>(tentatives & de & suicide)</td></tr>
<tr><td rowspan="2">Autolyse</td><td>(autolyse)</td><td rowspan="2">(?i)tentative\s+d'autolyse
(?i)autolyse</td></tr>
<tr><td>(tentative & d'autolyse)</td></tr>
</table>

	Autodestruction	(autodestruction)	(?i)autodestruction

Supplementary Table6. Dictionary used to detect risk factors

<table>
<tr><th>Risk factor (English)</th><th>Risk factor (French)</th><th>Regular expression used in the detection algorithm (French)</th></tr>
<tr><td rowspan="17">Sexual assault</td><td rowspan="17">Agression sexuelle</td><td>(?i)((agressions?)|(attaques?)|(atteintes?)|violences?|menaces?|outrages?)\s+(sexuel(le)?s?|sexistes?)</td></tr>
<tr><td>(?i)abus\s+sexuel</td></tr>
<tr><td>(?i)victime\s+d'abus</td></tr>
<tr><td>(?i)p[éeè]dophilie</td></tr>
<tr><td>(?i)\bviols?\b</td></tr>
<tr><td>(?i)attouchement</td></tr>
<tr><td>(?i)harc[èeé]lements?\s+sexuel</td></tr>
<tr><td>(?i)p[èeé]n[èeé]tration</td></tr>
<tr><td>(?i)p[èeé]n[èeé]tr[èeé]r</td></tr>
<tr><td>(?i)attentat\s+[àa]\s+la\s+pudeur</td></tr>
<tr><td>(?i)s[èeé]vice\s+sexuel</td></tr>
<tr><td>(?i)\babus[èeé]\b</td></tr>
<tr><td>(?i)cyber\s+agression\s+[àa]\s+caract[éeè]re\s+sexuel(le)?</td></tr>
<tr><td>(?i)((mutilation)|(traumatisme)|(l[éeè]sions?))\s+((g[éeè]nitale?)|(hym[éeè]n[éeè]ale))</td></tr>
<tr><td>(?i)(propos|comportements?)\s+[aà]\s+connotation\s+(sexuel(le)|sexiste)?</td></tr>
<tr><td>(?i)rapport\ssexuel\s((non\sconsenti)|forcé|(de\sforce))</td></tr>
<tr><td>(?i)gestes?\sd[eé]placés?</td></tr>
<tr><td rowspan="2">Domestic violence</td><td rowspan="2">Violence domestique</td><td>(?i)((tensions?)|(agressions?)|(violences?)|(conflits?)|(harc[èeé]lements?)|(brimades?)|(injures?)|tortures?)\s*((familiale?)|(dans\s+la\s+famille)|(((au)|(dans\s+le))\s+foyer)|(domestique)|(conjugale?)|(à\s+la\s+maison)|((par|avec)\s+(son|sa)\s+conjointe?)|(intrafamiliaux)|(intrafamiliale?))</td></tr>
<tr><td>(?i)conjugopathie</td></tr>
<tr><td>Social isolation</td><td>Isolement social</td><td>(?i)(isolement\ssocial)|(solitude)|(solitaire)|(mal\s+du\s+pays)|(z60\.?2)</td></tr>
</table>

<table>
<tr><td></td><td></td><td>(?i)(sentir\s+seule?)</td></tr>
<tr><td></td><td></td><td>(?i)((sentir|patiente?|enfant)\s+isolée?)</td></tr>
<tr><td></td><td></td><td>(?i)(sent\s+seule?)</td></tr>
<tr><td></td><td></td><td>(?i)pas\sde\sfamille</td></tr>
<tr><td></td><td></td><td>(?i)pas\sd.animal\sdomestique</td></tr>
<tr><td></td><td></td><td>(?i)isolement\sdans\sla\sclasse</td></tr>
<tr><td rowspan="4">Physical violence</td><td rowspan="4">Violence physique</td><td>(?i)(violences?|maltraitances?|s.vices?|agressions?|attaques?)\s+physiques?</td></tr>
<tr><td>(?i)r45\.?6</td></tr>
<tr><td>(?i)t74\.?1</td></tr>
<tr><td>(?i)\bbattue?\b</td></tr>
</table>

Document-classification algorithm

The document-classification algorithm consisted in detecting first all entities (i.e., terms) relative to SA in the discharge summary. The text around these entities was pre-processed (tokeniser, sentenciser). These entities were then passed to an entity-classification algorithm that used the context of the entity to determine whether it corresponded to the mention of a SA-caused hospitalisation or to something else. Indeed, a purely dictionary-based approach would have led to many false positive detections as it would not consider the context of SA mentions. In particular, mentions may be negated, formulated as a hypothesis, not relative to the patient or expressed as a reported speech. The document-classification algorithm classified the document as a true SA-caused hospitalisation if at least one entity was validated by the entity classification algorithm.

Entity-classification algorithm: case 1 - machine learning

The entity-classification algorithm classified each retrieved entity as a true or a false detection. A machine learning model was used therefore, that consisted in a single RoBERTa binary mono-label classification head on top of the eds-CamemBERT model that was itself fed by the output of a CamemBERT tokeniser.[4] eds-CamemBERT is a word embedding model that had been previously fine-tuned on 21 million French clinical documents of the clinical data warehouse.[5] The entity to be classified was fed to the model along with its context (a window of 35 words before the first word of the entity and 10 words after the last word of the entity). The eds-CamemBERT model provided an embedding vector of dimension 512 for each token. The embedding of the first token of each entity was fed into a classification layer. Our method differed from the original RoBERTa method as we classified the token of interest (i.e., not the <s> token as in the original implementation for sentence level classification task). Using a machine learning approach for entity classification in addition to the rule-based approach for entity recognition (regular expressions) led to an overall hybrid approach for text processing.

Entity-classification algorithm: case 2 - rule-based

As part of the sensitivity analysis, an alternative purely rule-based approach was also implemented for entity-classification. It relied on the use of the open-source *EDS-NLP* library dedicated to the development of rule-based NLP algorithms for the analysis of French clinical documents.[6] This library includes a dedicated detection pipeline for term modifiers (family, patient's history, reported speech, negation, hypothesis) and for the detection of dates. In our case, the detected dates were linked to the detected entity if they were in the same sentence and if the date did not correspond to the patient's birth date. If the mentioned date was at least 15 days before the start of the stay, the entity was classified as being part of the patient's history.

Risk factors detection

Each stay was classified as mentioning or not the five risk factors (RF) considered in this study.

- Social isolation, domestic violence, sexual violence and physical violence: the stay-classification algorithm for these RF followed the architecture of the rule-based stay-classification algorithm for SA but using another dictionary (Supplementary Table6) and considering only the negation and hypothesis modifiers to discard false positive detections.
- Suicide attempt history: a stay was classified as positive if at least one SA entity was detected in its discharge summary that was neither negated, relative to another experiencer, nor expressed in a hypothetical sentence and that was qualified as being part of the patient's history. Therefore, we used the same text-processing architecture than the rule-based algorithm for the detection of SA-caused stays (see Supplementary Figure3).

Development

Dictionary

The dictionaries (Supplementary Tables5-6) and the annotation guideline were initialised by asking a college of junior and senior psychiatrists coming from both pediatric and adult psychiatric units about their a priori knowledge of synonyms used to mention suicide attempts in clinical documents. This initial lexicon was expanded in families of keywords by data scientists to consider usual abbreviations and some typographic errors. It was then translated to the syntax of the preselection query engine. For example: *"tentative de suicide"* was expanded in {*"tentative de suicide"*,*"tentatives de suicide"*} and then translated to {(tentative & de & suicide) , (tentatives & de & suicide) }. The query engine was not sensitive to case, therefore all terms were expressed in lowercase. We also considered possible spelling errors regarding the accent marks, points and other syntactic markers. For instance, for the lexical variant *"tentative d'auto-strangulation"* we also considered *"tentative d auto strangulation"*. The textual criteria of Supplementary Table5 were finally concatenated with the OR (|) logic operator to form the final query. The objective was to increase the sensitivity of the first selection step (i.e., screening, see Supplementary Figure16).

We improved the dictionary using the results of the first annotation campaign on documents that had been pre-selected using the initial dictionary. In particular, we developed regular expressions in addition to the simple keywords used in the preselection query engine. These regular expressions were applied in the stay-classification algorithm and they could discard some false positive cases that were often encountered (e.g., *"ts en regression"* were *"ts"* stands for *"tissus sains"* -healthy tissue-). The first annotation campaign dedicated to the algorithm training consisted for each eligible document of the training set (i.e., any discharge summary containing at least one term of the dictionary) in annotating all the detected entities of SA or RF and in collecting keywords that were not already in the dictionary. All the documents were

automatically pre-annotated with the rule-based algorithm in order to facilitate the annotator's task.

Machine learning algorithm
The annotated dataset of 1571 SA entities was randomly split into a ML-training and a ML-development dataset (containing 1216 and 355 entities, respectively). The SA entity to be classified was fed into the model along with a context window (containing 35 words before the entity and 10 words after it). The objective of the model was to label the first token of each SA-entity to a binary value: 1 if the entity corresponded to a SA-caused hospitalisation, 0 otherwise. We optimised the cross-entropy loss function. The dropout rate was set to 0.1 during the training of the machine learning algorithm..
A simple hyperparameter search was done on the development set for the learning rate and the batch size. The search space for the learning rate was {1e-5, 2e-5, 3e-5, 5e-5} and for the batch size {16,32}. We used a grid search method and the combination with the best F1-score is used. Finally, the learning rate was set to 3e-5 and the batch size to 16.
We used the Adam optimise with the learning rate set to 3e-5, the weight decay to 0.1, the β1 to 0.9, the β2 to 0.98 and the ϵ to 1e-6. We trained the ML model during 10 epochs with a warmup of the learning rate during 2 epochs. The best epoch checkpoint regarding the F1 score of the development set was kept. We used the LambdaLR scheduler with default parameters.

Validation
When the algorithms were deemed satisfactory, they were frozen and their performances were assessed on the validation set.

- Validation of the main (hybrid) SA-detection algorithm: 162 stays detected as being caused by SA (85 and 77 for each period, pre- and post-pandemic) and that took place in one of the hospitals of the validation set were drawn randomly. Two clinicians conducted a chart review and labelled each stay as being a true positive or a false positive detection of a SA-caused hospitalisation.
- Validation of the RF-detection algorithms: for each RF, at least 40 stays (at least 20 for each period, pre- and post-pandemic) were drawn randomly among the set of stays detected as being caused by SA, that took place in one of the hospitals of the validation set and with a detected positive mention of the RF.
- Validation of the alternative (rule-based) SA–detection algorithm: we first applied the rule-based SA-detection algorithm on the stays that were classified as being caused by SA by the main hybrid algorithm and that had already been manually annotated. We computed $PPV_{main\ and\ RB}$ the predictive positive value of the rule-based algorithm on this dataset. This allowed us to reuse already-annotated data, but was biased towards stays detected by the hybrid algorithm. In order to remove this bias and complete the dataset with stays not detected by the hybrid algorithm, we drew randomly in the total dataset 40 additional records (24 and 16 for each pre- and post-pandemic periods) among those that were classified as being caused by SA only by the alternative rule-based algorithm. $PPV_{only\ RB}$ the PPV of the rule-based algorithm on this dataset was estimated by conducting a chart review. The overall PPV of the alternative algorithm was then estimated using the following equation:

$$eEq1: PPV = p_{main\ and\ RB} \times PPV_{main\ and\ RB} + (1 - p_{main\ and\ RB}) \times PPV_{only\ RB}$$

with $p_{main\ and\ RB}$ the probability of a record drawn randomly among all those that were classified as being SA-caused by the rule-based algorithm in the total dataset to be also

classified as SA-caused by the main, hybrid algorithm. In that case the 95% confidence interval was not computed as the Wilson method could not be applied.

The annotator accessed the last-edited discharge summary of the annotated visit. The inter-annotator agreement was measured by an annotation of approximately 10% of the stays by two annotators. The inter-annotator positive and negative agreements were [0.92;0.5] for SA detection, [1.0;1.0] for history of SA, physical violence, sexual violence, domestic violence and [1.0;-] for social isolation (i.e., no false positive detection was observed by the clinicians in the doubly annotated dataset). Three stays with annotator disagreement were re-annotated and corrected in the validation dataset.

Annotation guidelines

- *Suicide attempt*

Definition

We defined a suicide attempt (SA) as a self-directed potentially injurious behavior with any intent to die as a result of the behavior.[7]

General annotation guidelines

We applied the following rules to annotate SA in clinical documents:

- We discarded both the mentions of self-harm that did not explicitly indicated the intent to die (e.g., scarifications) and the mentions of suicide ideation;
- Suspicions of SA were not considered as SA;
- Intentional drug overdose or defenestrations were considered as SA even if the intentionality to die was not always explicitly stated as in these cases the intentionality is often implicitly meant;

Entity-level annotation guidelines

We completed the general guidelines to realise the entity-level annotation of the training dataset. We distinguished the annotation of each mention of the SA concept from its characterisation through the following attributes:

- Negation: if the patient or the clinical staff are denying or negating the suicide attempt (e.g., *"she never attempted suicide"*)
- Family: if the patient or the clinical staff are referring to the patient's close circle who attempted suicide (e.g., *"the patient's father attempted suicide"*)
- Hypothesis: if the mention of suicide attempt is expressed in an hypothetical sentence (e.g., *"the patient may have attempted suicide"*)
- History: if the mention is related to a previous suicide attempt that did not directly cause the hospitalisation. When dates are available, we consider that a suicide attempt occurring more than 15 days before hospitalisation did not directly cause it (e.g., *"the patient attempted suicide on January 1st, 2022"* for an hospitalisation on January 24th, 2022)
- Reported Speech: if the mention of suicide attempt is expressed by someone else than the clinician (e.g., *"the patient indicated that he attempted suicide"*)

We provide some examples to illustrate these guidelines:

- Example 1: a negative sentence mentioning SA
  - French: *"il ne s'agit pas d'une TS car la blessure est involontaire"*
  - English: "*it is not a SA because the injury is not voluntary*"
  - Annotation: *"positive"* SA concept and negation attribute
- Example 2: a mention detected by the algorithm that did not correspond to a SA
  - French: *"le patient prend ses médicament ts les 2 jours"*
  - English: *"the patient takes his medication every 2 days"*
  - Annotation: *"negative"* SA concept
- Example 3: a patient reporting a SA
  - French: *"Le patient nie que ce sont geste était une tentative de suicide»."*
  - English: *"The patient denies that he attempted suicide"*
  - Annotation: *"positive"* SA concept and reported speech attribute.

Stay-level annotation guidelines

We completed the general guidelines to realise the stay-level annotation of the validation dataset:

- A stay was labeled as a *"positive"* SA if the stay was caused by a suicide attempt realised by the patient less than 15 days before hospitalisation
- A stay was labelled as a *"positive"* SA if a SA occurred during hospitalisation. In that case the admission to hospital is not caused by SA, but the duration of the hospitalisation is augmented because of the SA and for the sake of simplicity we have therefore chosen to label the overall hospitalisation as SA-caused.
- When there was a contradiction in the annotated clinical document between the reason for hospitalisation and its conclusion, we considered the conclusion as the truth.

- *Risk factors*

Definitions

We started by defining the following two first risk factors:

- Social isolation: was defined as a lack of social contact. We underline that this definition was not equivalent to loneliness, that is a feeling. Social isolation is usually an observation of the clinician.
- History of suicide attempt: defined as a confirmed previous suicide attempt of the patient that did not directly lead to her hospitalisation.

We completed these risk factors by three additional risk factors related to violence:

- Sexual violence: we followed the World Health Organization (WHO) definition of sexual violence as being *"any sexual act, attempt to obtain a sexual act, unwanted sexual comments or advances, or acts to traffic, or otherwise directed, against a person's sexuality using coercion, by any person regardless of their relationship to the victim, in any setting, including but not limited to home and work."*.[8]
- Physical violence: we restricted the WHO definition of violence to its physical aspect and discarded violence against oneself, leading to the definition of physical violence as *"the intentional use of actual physical force against the patient that either results in or has a high likelihood of resulting in injury, death, psychological harm, maldevelopment or deprivation."*.[8]
- Domestic violence: we restricted the WHO definition of violence to violence exerted on the patient by members of her domestic environment: *"The intentional use of physical force or power, threatened or actual, exerted on the patient by a member of her family or household members that either results in or has a high likelihood of resulting in injury, death, psychological harm, maldevelopment or deprivation."*.[8]

Annotation guidelines

We applied the following rules to annotate risk factors at the stay level during the validation campaign:

- Social isolation: clinical documents sometimes mentioned aspects that could be interpreted as social isolation even if their qualification as a risk factor was not explicitly stated. In that case we nevertheless annotated the stay as mentioning social isolation (e.g., when the clinician mentioned the absence of domestic animals).
- History of suicide attempt: when the date of a previous SA was available, we considered that it was part of the patient's history if it occurred at least 15 days before the hospitalisation. When the date of the previous SA was not explicitly mentioned, if it was clearly stated that SA was not the direct cause of the hospitalisation, we nevertheless annotated it as a history of suicide attempt.
- Sexual violence: some ambiguous expressions that are mostly referring to sexual violence were labelled as positive sexual violence (e.g., French *"gestes déplacés"*, English: *"inappropriate gesture"*).

- Physical violence: we did not label mentions of sexual violence as being also mentions of physical violence. A clinical document mentioning a rape could for instance be labeled as *“positive”* for the sexual violence risk factor but *“negative”* for the physical violence risk factor.
- Domestic violence: if a violence was exerted on the patient by her family, we labelled it as domestic violence even if we did not know whether they both lived at the same place

We underline that a stay could be labelled “positive” to more than one risk factor as they were not mutually exclusive.

References

1. Jollant, F. *et al.* Hospitalization for self-harm during the early months of the COVID-19 pandemic in France: A nationwide retrospective observational cohort study. *Lancet Reg. Health Eur.* **6**, 100102 (2021).
2. Murphy, S. N. *et al.* Serving the enterprise and beyond with informatics for integrating biology and the bedside (i2b2). *J. Am. Med. Inform. Assoc. JAMIA* **17**, 124–130 (2010).
3. Downs, J. *et al.* Detection of Suicidality in Adolescents with Autism Spectrum Disorders: Developing a Natural Language Processing Approach for Use in Electronic Health Records. *AMIA Annu. Symp. Proc. AMIA Symp.* **2017**, 641–649 (2017).
4. Liu, Y. *et al. RoBERTa: A Robustly Optimized BERT Pretraining Approach*. http://arxiv.org/abs/1907.11692 (2019) doi:10.48550/arXiv.1907.11692.
5. Dura, B. *et al. Learning structures of the French clinical language:development and validation of word embedding models using 21 million clinical reports from electronic health records*. http://arxiv.org/abs/2207.12940 (2022) doi:10.48550/arXiv.2207.12940.
6. Dura, B. *et al.* EDS-NLP: efficient information extraction from French clinical notes. (2022) doi:10.5281/zenodo.6818507.
7. Cousien, A., Acquaviva, E., Kernéis, S., Yazdanpanah, Y. & Delorme, R. Temporal Trends in Suicide Attempts Among Children in the Decade Before and During the COVID-19 Pandemic in Paris, France. *JAMA Netw. Open* **4**, e2128611 (2021).
8. Krug, E., Dahlberg, L., Mercy, J., Zwi, A. & Lozano, R. *World report on violence and health*. http://apps.who.int/iris/bitstream/handle/10665/42495/9241545615_eng.pdf?sequence=1 (2002).
